# From *Diospyros kaki* L. (Persimmon) Phytochemical Profile and Health Impact to New Product Perspectives and Waste Valorization

**DOI:** 10.3390/nu13093283

**Published:** 2021-09-20

**Authors:** Rosa Direito, João Rocha, Bruno Sepodes, Maria Eduardo-Figueira

**Affiliations:** 1 Research Institute for Medicines (iMed.ULisboa), Faculdade de Farmácia, Universidade de Lisboa, 1649-004 Lisbon, Portugal; jrocha@ff.ulisboa.pt (J.R.); bsepodes@ff.ulisboa.pt (B.S.); efigueira@ff.ulisboa.pt (M.E.-F.); 2 Department of Pharmacy, Pharmacology and Health Technologies, Faculdade de Farmácia, Universidade de Lisboa, 1649-004 Lisbon, Portugal; 3 Department of Pharmaceutical Sciences and Medicines, Faculdade de Farmácia, Universidade de Lisboa, 1649-004 Lisbon, Portugal

**Keywords:** *Diospyros kaki* L., phenolic compounds, proanthocyanidins, antioxidant, sustainability, new products, food crisis, diet, sustainable nutrition, byproduct valorization

## Abstract

Persimmon (*Diospyros kaki* L.) fruit’s phytochemical profile includes carotenoids, proanthocyanidins, and gallic acid among other phenolic compounds and vitamins. A huge antioxidant potential is present given this richness in antioxidant compounds. These bioactive compounds impact on health benefits. The intersection of nutrition and sustainability, the key idea behind the EAT-*Lancet* Commission, which could improve human health and decrease the global impact of food-related health conditions such as cancer, heart disease, diabetes, and obesity, bring the discussion regarding persimmon beyond the health effects from its consumption, but also on the valorization of a very perishable food that spoils quickly. A broad option of edible products with better storage stability or solutions that apply persimmon and its byproducts in the reinvention of old products or even creating new products, or with new and better packaging for the preservation of food products with postharvest technologies to preserve and extend the shelf-life of persimmon food products. Facing a global food crisis and the climate emergency, new and better day-to-day solutions are needed right now. Therefore, the use of persimmon waste has also been discussed as a good solution to produce biofuel, eco-friendly alternative reductants for fabric dyes, green plant growth regulator, biodegradable and edible films for vegetable packaging, antimicrobial activity against foodborne methicillin-resistant *Staphylococcus aureus* found in retail pork, anti-*Helicobacter pylori* agents from pedicel extracts, and persimmon pectin-based emulsifiers to prevent lipid peroxidation, among other solutions presented in the revised literature. It has become clear that the uses for persimmon go far beyond the kitchen table and the health impact consumption demonstrated over the years. The desired sustainable transition is already in progress, however, mechanistic studies and clinical trials are essential and scaling-up is fundamental to the future.

## 1. Introduction

Persimmon (*Diospyros kaki* L.) is a member of the Ebenaceae family and is a very popular and important fruit in East Asian countries, being widely produced in China, South Korea, and Japan [1]. The name “persimmon” (Diospyros) originates from the Greek *dióspuron*, which means “food of Zeus”, while “kaki” comes from the Japanese kaki (柿) [2]. Recently, persimmon’s popularity has grown outside its traditional region of production (China, Japan, and Korea), growing into an encouraging crop in Brazil and in some Mediterranean countries such as Italy, Spain, and Portugal. The cultivation of persimmon in Europe is limited to the proximity to the Mediterranean Sea [3]. China is the largest persimmon producer, producing around 3.03 million tons, followed by Spain with 400,000 tons, South Korea with 300,000 tons, Japan with 225,000 tons, and Brazil with 182,000 tons. In Portugal, for example, there are some dedicated orchards in the south of the country such as in the Algarve region, although most persimmons come from trees scattered throughout the central and northern regions of country. The world production of persimmon is around five million tons, corresponding to 0.75% of total fruit production [4].

The persimmon tree belongs to the genus Diospyros, Ebenaceae family, and has four commercially important species, of which the most representative is *Diospyros kaki* (Japanese persimmon) having, however, other equally relevant species such as *Diospyros virginiana*, *Diospyros oleifera*, and *Diospyros lotus* [2,5].

Persimmon is a spherical fruit with a color that ranges from reddish to yellow according to carotene content, the pulp is a viscous orange-red and somewhat rough, depending on the content of tannins. The pulp, regardless of the variety, consists mainly of mucilage and pectin, which is responsible for its characteristic appearance [6].

Persimmon is a seasonal fruit: it is only available in its fresh form for a short-time window throughout the year. In Europe, persimmons can be purchased in late autumn and early winter, from September to early December [7,8].

**Figure 1:**
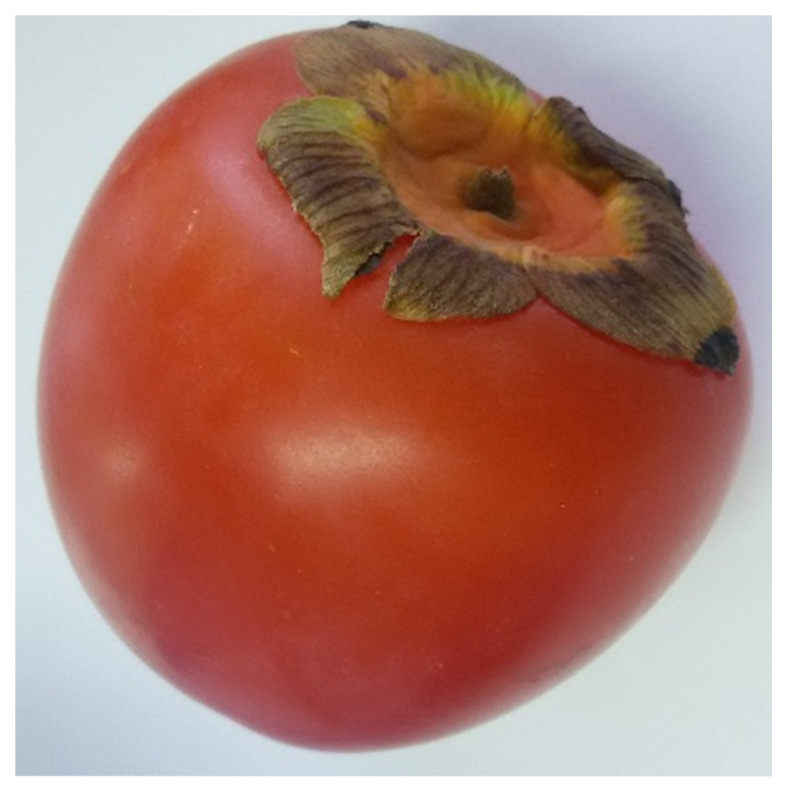
Persimmon, *Diospyros kaki* L. fruit from Algarve, Portugal.

In several studies, it has been described that the concentration of nutrients and other functional components of persimmon is higher in the skin than in the pulp [9]. However, the skin of the fruit is typically discarded when it is consumed with a spoon (Figure 1) when it is very ripe, or during the drying process of the persimmon [1]. This process of fruit preservation is widely used in China and Japan, given the large quantities produced, its usage in a high number of culinary recipes, and the need to preserve it in significant quantities in a long-lasting and safe way [10].

Persimmon, in addition to its nutritional value, has been used in traditional Chinese medicine for its beneficial effects in health, namely against hypertension, hemorrhages [11], in maintaining body temperature, and slowing oxidative processes in general [12,13], due to their diuretic effect, in diabetes, and atherosclerosis [14]. It has also been used to improve the function of the lungs, stomach, spleen, and intestines and used in the prevention and treatment of diseases such as sore throat, thrush, and insomnia [15].

Several studies have shown that persimmon has anti-tumor properties [16,17], prevents dyslipidemia [18], has anti-hypercholesterolemic, antioxidant effects [19,20], and antidiabetic effects [21]. These health benefits have been associated with its richness in antioxidants including vitamins, phenolic compounds, and carotenoids [15,22].

The EAT-*Lancet* Commission gathered all accessible nutritional and environmental data to build the first global benchmark diet able to sustain health and protect the planet [23]. The intersection of nutrition and sustainability is the key idea behind the EAT-*Lancet* diet. This report supports the concept of building a diet that could improve human health and decrease the global impact of food-related health conditions such as cancer, heart disease, diabetes, and obesity. The EAT-*Lancet* commission finally recommended a ‘flexitarian’ diet that covers a range of food groups, even though it also suggested vegan and vegetarian options. Plants shape the substance of the commission’s flexitarian diet, which suggests the daily consumption of about 230 g of whole grains, 200 g of fruit, 300 g of vegetables, and 125 g of plant-based protein-rich foods such as nuts, dry beans, and lentils [24,25]. Existing diets diverge considerably from the EAT-*Lancet* targets [23].

Taking this perspective into consideration, a review focusing on persimmon fruit was performed with the aim of looking into the health impact of its consumption and the nutritional and phytochemical profile.

### 1.1. Persimmon Composition

#### 1.1.1. Nutritional Characterization

Persimmon has a pretty good nutritional value: it contains many compounds such as different sugars, starch, organic acids and amino acids, proanthocyanidins (PACs), flavonoids, carotenoids, triterpenoids and fatty acids [26,27,28,29,30], proteins, and vitamins A, B6, B12, C, and D [31].

According to some authors, persimmons contain around 80.3% water, 0.58% protein, 0.19% total lipids, 18.6% total carbohydrates and some minerals (calcium, potassium, magnesium, sodium, iron, zinc, copper, manganese, phosphorus, selenium), up to 1.48 g of total dietary fiber, and 7.5 mg of ascorbic acid [32,33].

In the following table (Table 1), the general nutritional composition of persimmon is presented.

It is documented that persimmons have a content in the order of 12.5 g of sugar per 100 g of fruit, with fructose, glucose, and sucrose present in greater quantities [26,36,37,38,39], and containing smaller quantities of maltose, xylose, arabinose, and glucuronic acid [40]. The amounts of these sugars may, however, vary significantly between cultivars and their degree of ripeness [40,41]. In the study performed by Ryu et al. on the composition of the persimmon, where they used various techniques combined with NMR, it was possible to demonstrate that glucose and fructose are present in the aqueous extract of the fruit juice in much greater quantity than sucrose [42]. According to Giordani et al., extraction methods significantly affect the sugar composition of the extracts obtained due to the activity of invertase, which degrades sucrose during extraction [43]. The low percentage of sucrose observed by Ryu et al. [42] may in fact be due to the invertase activity in the pulp of the persimmon.

Having an important mineral composition, consumption of one piece of this fruit (200–400 g) can provide 1–10% of the recommended daily allowance (RDA) for calcium, 1–30% for copper and potassium, 1–15% for iron and magnesium, up to 1% sodium, and up to 4% zinc [44].

Persimmons are a good source of carotenoids, which even though they are mostly found in the skin [45], the pulp still contains large amounts of these compounds, responsible for the color of the fruits and whose amount increases as the fruit ripens [26,46]. Persimmons have 2 mg of carotenoids/100 g of fresh fruit (FF) [47], which corresponds to 22% of the RDA of vitamin A [35]. This group of compounds has been implicated in the reduction in degenerative diseases in humans due to their antioxidant capacity and free radical uptake properties [48,49,50]. Since its main carotenoids are β-cryptoxanthin (193 μg/100 g), β,β-carotene (113 μg/100 g FF), and β-carotene (30 μg/100 g FF) [43], it is generally β-cryptoxanthin that is found in greatest quantity [51].

Esters of carotenoid fatty acids (*Diospyros kaki* THUNB.), lycopene, β-carotene, ester of zeaxanthin di-myristic acid, and mono-myristic acid ester of β-cryptoxanthin have all been characterized in the persimmon peel, while a small quantity of palmitoleic acid in the fatty acid and oleanolic acid fraction was found [52,53].

With regard to vitamin C, the broad variations in its content present in persimmons as reported [43,54] can be explained through environmental factors, by the fruit’s inherent qualities, by its time of harvest, and the general conditions of the crop. These elements have an impact on the daily reference intake of vitamin C (80 mg/day), which covers the eating of this fruit. As such, one unit of persimmon fruit (average weight = 263.32 g) can satisfy anywhere between 15.21% and 33.74% of the daily reference intake of vitamin C [55], or as demonstrated by Rao and colleagues, a persimmon can supply around 46% of this vitamin’s daily requirements [56].

The levels of vitamin C in cultivars that are non-astringent are considerably greater (10 times) than in cultivars that are astringent [43]. It should be referenced that persimmon fruit has two types of vitamin C: L-ascorbic acid and its oxidized product, L-dehydroascorbic acid. Although both chemical species are important compounds thanks to their antioxidant activities, they do not have the same activity, since L-ascorbic acid is more active than the oxidized form [57]. About 2/3 of the total vitamin C in the persimmon is available as L-ascorbic acid [43].

In persimmon extracts, organic acids (malic, oxoglutamic, citric, succinic, and aspartic), amino acids (gamma-aminobutyric acid (GABA), alanine, arginine, valine, leucine, and isoleucine), fatty acids (palmitoleic acid methyl ester, myristic acid, and linoleic acid) [40,52], vitamins (trigonelline and ascorbic acid), α-tocopherol, oleanolic acid, betulinic acid, β-sitosterol, and uridine have also been identified [40].

It is also known that organic acids occur naturally in food as a result of metabolic processes, namely in the metabolism of abscisic acid, and are related to the tolerance to environmental stress in plants [58], the main ones in persimmon being malic and citric acids [42,59]. In the studies by Senter et al. with persimmons, these acids were quantified, where malic acid was the predominant acid in all cultivars studied by the authors, followed by citric [60]. The amounts of malic acid increased with maturation and those of citric decreased [41].

The amino acid *L*-tryptophan has been identified in recent studies in the aqueous extract of persimmon when analyzed based on NMR techniques [42]. With HPLC-DAD-ESI-TOF/MS technology applied to persimmon juice, the precursor ion m/z 203 was also identified as the amino acid *L*-tryptophan [38]. In studies on the characterization of persimmon juices using HPLC-DAD-ESI-TOF/MS methodology by Jiménez-Sánchez et al., various proteins and derivatives were detected. Precursor ions with m/z 164, 180, and, 203 were identified as *L*-tyrosine, *L*-phenylalanine, and *L*-tryptophan. These authors reported the detection of precursor ions m/z 395 and 383, which they considered as peptides, with the possibility of being *L*-lysine, *L*-serine, *L*-tyrosine/*L*-serine, *L*-proline, for the first and *L*-lysine, *L*-trionine, and *L*-histidine for the second [38,59].

Domínguez Díaz and colleagues characterized the ‘Rojo Brillante’ persimmons (*Diospyros kaki* L.), Protected Designation of Origin (PDO) ‘Ribera del Xúquer’ with regard to the existence of fiber with a total content of 2.38–4.99 g/100 g FW (vaguely greater for soluble fiber); vitamin C in the range of 4.62–10.25 g/100 g FW (primarily as dehydroascorbic acid); carotenoids (with lycopene as primary, in 26.76–51.10 μg/100 g FW, followed by β-carotene, in 10.07–20.50 μg/100 g FW, and neoxanthin, violaxanthin and β-cryptoxanthin as minor compounds); and mineral elements (Ca, Cu, Fe, K, Mg, Mn, Na, and Zn). With this information in mind and taking into consideration the scientific results surrounding this fruit showing the richness of persimmon fruit in bioactive compounds such as carotenoids, fiber, and vitamin C, not to mention the micro- and macro-minerals with significant health-furthering properties, the authors propose that the use of persimmon fruit and its bioactive components may be a solid approach to improving the population’s health level globally. However, even though the consumption of persimmons brings important health benefits, to date, there are no specific health claims authorized for this fruit. As such, Domínguez Díaz and co-authors suggested that Persimon^®^ (the registered trademark for the ‘Rojo Brillante’ variety) fruit is in theory capable of demonstrating two nutrition allegations: “sodium-free or salt-free” and “source of fiber”. Persimon^®^ fruit ought to have 3 g at a minimum of total dietary fiber/100 g of edible portion or at least 1.5 g per 100 kcal for the use of the “source of fiber” nutrition assertion. Given that the total dietary fiber mean values of all analyzed batches (2017 and 2018 seasons) were greater than the necessity of 1.5 g of fiber/100 kcal, it could be feasible to present the nutrition assertion “source of fiber” for Persimon^®^ fruits. The nutrition claim “sodium-free or salt-free” might also be used for Persimon^®^ given that its sodium content is inferior to the requirement of 0.005 g of sodium. These results may prepare the conditions for considering natural food products as candidates for the use of approved nutrition claims such as those used for this or other persimmon varieties [61], similarly to what has also been described for raspberry fruit (*Rubus idaeus* L.) [62]. It should also be noted that it has also been explained that the recommended daily intake of fresh persimmon should be around 100–150 g [43].

#### 1.1.2. Phenolic Compounds

Phenolic compounds (PCs) can be found in a variety of foods available to the human diet including vegetables, fruits, beverages, herbs, and spices, many of which have been used empirically by humanity for centuries, namely in traditional medicine [63]. Fruits and vegetables, and their products, are among the foods richest in these compounds.

The persimmon is rich in phenolic compounds already identified as bioactive [36,37,40]. Much like carotenoids, PCs are also more abundant in the peel when compared to the pulp [64].

There is an interest in these compounds, which is mainly due to their antioxidant capacity (reaction with free radicals and chelation of metals) and possible beneficial implications for human health such as prevention and co-adjuvants in the treatment of various types of cancer, cardiovascular diseases, and other pathologies [65,66,67]. In addition to their antioxidant capacity, phenolic compounds have many other characteristics such as anti-atherogenic, anti-tumor, and anti-inflammatory effects, which cannot be described based only on their antioxidant properties [68].

Persimmons are rich in flavonoids, terpenoids, naphthoquinones, saponins, and condensed tannins [69]. In addition to the low molecular weight phenols present in the edible part of the fruit, there are phenolic acids such as gallic acid and its glycoside and acyl derivatives, glycosides of p-coumaric and vanillic acid, caffeic, chlorogenic acids, and several different di-C-hexoxide flavones [70]. It is worth noting that the following flavan-3-ol monomers were detected in persimmons: catechin, epicatechin, and epigallocatechin [71]. It was also through the combination of several analytical techniques that it was possible to identify myricetin as the terminal unit of the most common flava-3-ois (catechin and epigallocatechin-3-*O*-gall) [72].

In their work, Chen, Fan, and colleagues determined the content of individual and total phenols (epigallocatechin, epicatechin, catechin, chlorogenic acid, gallic acid, and caffeic acid) and compared them with those of other fruits such as apples, grapes, and tomatoes. They then concluded that persimmon was the fruit with a higher content of total phenolic compounds (about 170 mg/100 g dry weight) when compared to grapes (about 100 mg/100 g dry weight), apple (about 40 mg/100 g dry weight), and tomato (about 20 mg/100 dry weight) [15]. The content of total phenolic compounds in persimmons was eight times greater than that of tomatoes, which is in line with the high antioxidant capacity of the persimmon extract [15]. Gorinstein and colleagues also obtained similar results, showing that the content of total phenolic compounds in persimmons was greater than that of apples [9].

Total phenolic content dosages in persimmons as reported by available literature differed extensively. Total phenolic compounds were registered to be in the range of 12.7 and 29.5 mg of GAE/100 g of fresh weight (FW) [26]. Other researchers have registered soluble polyphenol concentrations to vary in the range of 1.3 mg to 1.550 mg/100 g FW of total phenolic compounds as well for the same astringent Triumph cultivar [9,73]. As a matter of fact, if we consider the work done by Jang and co-authors performed on homogeneous samples of the non-astringent cultivar Fuyu, the smallest concentration of soluble polyphenols was 454 mg GAE/100 g FW with ethanol extraction, about half the maximum concentration determined through extraction with water (860 mg GAE/100 g FW) [37]. Denev and colleagues determined 916.8 mg GAE/100 g FW [74], and Direito and colleagues determined 641 ± 51.96 mg GAE/100 g FW [75]. The inconsistency detected in these assays can be illuminated by considering the edaphoclimatic conditions, analysis of distinct opossum cultivars, and maturation phases, even when the fruit is more suitable for eating. Additionally, the various extraction methods that were used as well as the analytical methods of the protocols may have had an impact on the results [43].

Through an improved extraction procedure, together with an ultraperformance liquid chromatography coupled with Q-TOF mass spectrometry (UPLC-Q-TOF-MS) platform, Esteban-Muñoz and colleagues characterized the PC composition of the two varieties of persimmon, Rojo Brillante and Triumph. The phenolic composition of the pulp of these two varieties was shown to have hydroxycinnamic and hydroxybenzoic acids, tyrosols, dihydrochalcones, hydroxybenzaldehydes, flavonols, flavanols, and flavanones. According to this team’s work, an overall amount of 31 compounds were detected, while 17 compounds were quantified. The prevalent phenolic compound observed in the Rojo Brillante type (0.953 mg/100 g fruit pulp) was gallic acid, while the concentration of p-hydroxybenzoic acid was greater in the Triumph variety (0.119 mg/100 g fruit pulp). The results demonstrated that the Rojo Brillante type had greater amounts of PCs than the Triumph type [20]. The concentrations of the phenolic compounds (caffeic acid, catechin, chlorogenic acid, epicatechin, fisetin, ferulic acid, gallic acid, p-coumaric acid, and protocatechuic acid) were determined by the ultra-high performance liquid chromatography (UHPLC-DAD) method (Table 2). In an aqueous persimmon fruit extract, gallic acid was found to be the most plentiful phenolic compound (2.794 ± 0.263 mg/100 g FW) detected [75], which is also in accordance with Pu and colleagues who found a concentration of 2.789 mg/100 g FW in the variety *D. kaki var. silvestris* M [76]. Nevertheless, Veberic and co-authors reported 2.43 ± 0.215 mg/100 g FW [26]. According to the work undertaken by Esteban-Muñoz et al., caffeic acid was the second most profuse hydroxycinnamic PC with 0.078 and 0.046 mg/100 g for the Triumph and Rojo Brillante varieties, respectively, levels that are consistent with those determined in other varieties of persimmon [76,77]. The concentrations of the three hydroxycinnamic acids (caffeic acid, ferulic acid, and chlorogenic acid) in the study done by Direito and colleagues were below 0.1 mg/100 g FW, except for the chlorogenic acid content, as was illustrated by Pu and colleagues [76]. The chlorogenic acid concentration in the work presented by these authors was 0.171 ± 0.016 mg/100 g FW *D. kaki* extract [75]. Published results of 0.274 mg/100 g FW in the variety of *D. kaki var. silvestris* M and 0.145 mg/100 g FW in the variety of *D. kaki cv.* Xingyangshuishi have also been found [76]. These same authors were not able to discover p-coumaric acid in five out of the six genotypes that were evaluated. The only genotype where p-coumaric acid was found was in *D. kaki var. silvestris* M, with a value of 0.048 ± 0.004 mg/100 g FW [76], roughly less than twice the concentration reported by Direito and colleagues of 0.097 ± 0.004 mg/100 g FW [75], in accordance with the concentration reported by Esteban-Muñoz and colleagues, where p-coumaric acid was the hydroxycinnamic acid with the greatest levels in both persimmon varieties (not statistically different) with ranges from 0.088 to 0.113 mg/100 g FW [20]. According to these authors’ work, protocatechuic acid had a concentration of 0.013 ± 0.010 mg/100 g in the Rojo Brillante variety, which was greater than those attained for the Triumph sample (0.004 mg/100 g), which is in agreement with the concentration reported by Direito and colleagues (0.005 mg/100 g FW) [75]. Greater concentrations were observed in various persimmon extracts acquired with various solvents [77], but the use of a HPLC equipped with ECD (electron capture detector) detection might overemphasize the concentration, and lower concentrations have also been described [75]. Esteban-Muñoz et al. identified ellagic acid for the first time in persimmon fruit in the Rojo Brillante variety (0.327 ± 0.173 mg/100 g fruit pulp), the variety of persimmons that had a greater concentration of ferulic acid than that found in the Triumph variety (0.011 vs. 0.008 mg/100 g). These concentrations are consistent with (or slightly lower than) those described for different persimmon varieties by other authors [75,76,78].

Compared with hydroxycinnamic acids, the extent of hydroxybenzoic acids was higher [20,75], but the (+)-catechin and (−)-epicatechin contents reported by Direito et al. were lower than previously reported values [79,80]. The same situation occurred for the concentration of the flavone fisetin [72]. Nevertheless, the various stages of maturation and the various cultivars may possibly lead to a variability in the results [43]. The diversity of small phenols in pulp extracts reported to date is, however, surprisingly scarce, being normally restricted to three to ten components (derived mainly from cinnamic acid), depending on the variety being assayed [43]. Thus, it can be concluded that the profile of low molecular weight phenols, for example, in the pulp of the persimmon, still remains to be determined [20,70].

Catechins (flavan-3-ol) are the main flavonoids found in persimmons, which can offer potential benefits to human health, given that they are related to several physiological functions including a protective role against diseases associated with oxidative stress and have antimutagenic and anticarcinogenic capabilities [71]. Persimmon extracts also showed apoptosis-inducing activity of Molt 4B leukemic cells [81].

Procyanidins consisting of (epi)catechin and (epi)gallocatechin together with free phenolic acids in extracts of different varieties of Japanese persimmon have been reported in the literature [71]. However, Sentandreu and colleagues found no evidence of their presence in their tested samples [70]. Zang and colleagues described the presence of the precursor ion m/z 289, which may be -(−)epicatechin [82] and with a fragmentation spectrum characteristic of this type of compound (m/z 109, 123, 125, 205, and 245) [38]. Epicatechin and flavone di-C-hexoside were, however, identified by Maulidiani and colleagues [59].

Quercetin was measured in comparable quantities in the Triumph and Rojo Brillante varieties in the range of 0.005–0.007 mg/100 g fruit pulp. Even though these values are lower than those described for other varieties of persimmon [76], some authors did not find any quercetin in persimmons [78]. Maulidiani and colleagues identified quercetin and kaempferol derivatives as the major flavonoids in persimmon where the four derivatives of quercetin including quercetin 3-(2′′-galloylglucoside), quercetin 3-*O*-glucoside and its isomer and quercetin aglycone were also identified. Kaempferol-3-*O*-glucoside and its isomer, kaempferol aglycone and kaempferol 3-(2′′-galloylglucoside) were likewise also identified [59].

Persimmons accumulate a large amount of condensed tannins (or proanthocyanidins, PACs) in vacuoles of specific cells called “tannin cells” during the development of the fruits, which are responsible for the astringency, which is a sensation of dryness or “tie the mouth” due to the clotting of oral proteins [83]. As maturation progresses, tannin content decreases and the risk of bitter taste of astringent varieties also decreases [84]. However, the greater or lesser astringency of all cultivars decreases during ripening, not only because of the decrease in tannin content, but because the soluble tannins turn into insoluble forms [85]. Astringent cultivars continue to have a significant amount of soluble tannins even when ripe [51].

Gu and colleagues used the HPLC method coupled with electrospray ionization mass spectrometry (ESI-MS) to determine the fractions of condensed tannins of high molecular weight that constitute the persimmon, which permitted them to verify that the antioxidant activity of high molecular weight tannin fractions was significantly higher than that of low molecular weight tannins and that of proanthocyanidins in grape seeds. This led the author to consider that condensed high molecular weight tannins were the largest antioxidant present in persimmon pulp [13]. As studied by Suzuki and colleagues, the highest proanthocyanidin content was found in astringent varieties [71]. Proanthocyanins diminished proportionately throughout maturation from 540.2 mg CE/100 g FW to 90.2 mg CE/100 g FW [74]. In the study by Direito and colleagues, proanthocyanidin content was 744 ± 8.6 mg CE/100 g FW [54].

In the study of the structure of the proanthocyanidins present in the persimmon, it was found that they consist essentially of (epi)gallocatechin-3-*O*-gallate, (epi)catechin-3-*O*-gallate, and epicatechin [13,72]. The molecular weight distribution of the condensed tannins of high molecular weight was determined as being in an interval between 1.16 × 10^4^ Da and 1.54 × 10^4^ Da [13], and Li and colleagues described between 7 and 20 kDa (degree of polymerization 19–47), but sizes estimated by gel permeation chromatography were 50–70% smaller [72].

Tyrosol was found at similar levels in both varieties of persimmon (Rojo Brillante and Triumph), 0.020 ± 0.017 mg/100 g fruit pulp for Triumph and 0.038 ± 0.021 mg/100 g fruit pulp for Rojo Brillante studied by Esteban-Muñoz and colleagues, but no other studies discussing tyrosol have been found to date [20].

Triterpene and fatty acids are detected in different amounts in all cultivars: Fuyu, Triumph, Krembo, Jiro, Romang, and Taishu. Spathodic acid, a pentacyclic triterpenoid, rotungenic acid, and oleanolic acid were identified based on the molecular ion at m/z 455.3532 [59]. Spathodic, barbinervic, and rotungenic acids, which were only found in Krembo, can be considered as the chemical markers of the Krembo cultivar. L-malic and citric acids were also found in all cultivars. Interestingly, pantothenic acid was only detected in the Krembo persimmon. According to analyses, monogalloyl-hexoside was only found in Krembo and Triumph, while gallic acid was found in Krembo, Triumph, and Jiro cultivars. Quercetin and kaempferol were found in the Fu and Ji varieties, while its glycosidic forms were mostly found in Fuyu, Krembo, Triumph, and Jiro. Fructose was found in all cultivars, while glucuronic acid was found in the Krembo variety and had 23 unique compounds, while Fuyu and Taishu varieties had only eight and two unique compounds, respectively. All three cultivars shared the same 15 compounds, which were mostly sugars, amino acids, and fatty acids [59].

Two benzoquinone derivatives were also identified based on their molecular ions at m/z 137.0233 and 293.1760, the 2-methoxy-1, 4-benzoquinone, and embelin, respectively. Embelin is a dihydroxy-1,4-benzoquinone that has an undecyl group substitution at position C3 [59].

### 1.2. Persimmon Biological Activity

Persimmon (*Diospyros kaki* L.) is a nutritious fruit with strong antioxidant activity [36], especially due to its richness in phenolic compounds, vitamin C, and carotenoids [26,37,47,52,86,87] responsible for the reduction in oxidative damage and, consequently, with activity in associated diseases such as cardiovascular diseases and diabetes mellitus. In addition, there is evidence that persimmons or their functional constituents such as proanthocyanidins can help in cases of hyperlipidemia and hyperglycemia [47,88,89,90]. According to many authors, this fruit has preventive effects on the arteriosclerotic process [9,36,73,91,92] and prevents metabolic syndrome [93] (Table 3).

When ROS is prevalent, if its production is excessive or the antioxidant systems are inadequate, oxidative stress is the result. From a medical point of view, oxidative stress can lead, for example, to atherosclerosis, inflammation, or cancer [94]. ROS/RNS is associated with the pathogenesis of cancer, atherosclerosis, cardiovascular disease, hypertension, diabetes mellitus, ischemic/reperfusion injury, neurodegenerative diseases (e.g., Parkinson’s disease and Alzheimer’s disease), rheumatoid arthritis, and aging [95]. Due to the association of reactive oxygen species with cancer and cardiovascular diseases, antioxidants have been considered a promising route for the prevention and treatment of these diseases, especially considering the links observed between diets rich in fruits and vegetables, and reducing the risks of developing cancer [96]. The phenolic compound quercetin, for instance, has been described as being able to effectively recycle vitamin E and has also been registered as being able to decrease cell damage caused by oxidation, inflammation, and tumorigenesis [97,98,99,100,101,102].

Compounds such as epigallocatechin, epicatechin, catechin, chlorogenic acid, gallic acid, caffeic acid [15], carotenoids [103], procyanidins [13,71], and ascorbic acid are the main antioxidants found in persimmon. The persimmon’s phenolic compounds are 20 times more potent in vitro than the classic antioxidant, vitamin E [104]. The elevated antioxidant potential of phenolic acids has been verified by several authors [36,73]. These are linked to a variety of physiological functions, which includes a protective role against diseases related to oxidative stress, antimutagenic, and anticarcinogenic capabilities [17,54,71].

Phenolic compounds have been known as powerful antioxidants through their capacity to capture and trap free radicals and sequester oxidizing agents, achieving better antioxidant capacity than vitamin C and vitamin E [105,106]. Given their chemical structure, phenolic groups can accept an electron or a proton, forming relatively stable phenolic radicals, preventing chain oxidation reactions in cellular compartments [106], but they can also alter the expression of multiple genes that encode enzymes with antioxidant function and cell signaling change [107].

An alternative mechanism may be that the phenolic components of the diet may increase the potential of endogenous antioxidant defenses and thus modulate the cellular redox state, both under normal conditions and during pathophysiological processes [68].

In summary, the demand for natural antioxidants has increased [108], resulting from the need to increase the endogenous antioxidant capacity via ingestion of foods rich in antioxidant compounds in order to cancel or reduce one of the risk factors in the development of chronic diseases. The chemical composition, in conjunction with in vivo and in vitro studies, indicates a relevant role of the persimmon in protecting against free radicals and in preventing certain human diseases [17,19,43,109,110].

#### 1.2.1. Proanthocyanidins (PACs) in Obesity and Lipidic Metabolism 

Several studies have shown the association of the persimmon’s procyanidins with various biological functions such as antioxidant activity [37,116,122], anti-inflammatory activity [17,123], antimicrobial [124,125,126], hypolipidemic [19,114,127,128], antidiabetic [21], and decreased risk of atherosclerosis [31,73,111].

The hypolipidemic effects of high molecular weight tannins were investigated by Zou et al., supplementing the diet of male Sprague–Dawley mice that consumed a 2% high-cholesterol diet and treated with different doses of high molecular weight tannins or without them, for nine weeks. High molecular weight tannins effectively reduced serum and liver triglycerides, total cholesterol, and LDL, while increasing serum and liver HDL. A treatment of 100 mg high molecular weight tannins/kg of body weight per day can significantly increase the activity of serum lecithin cholesterol acyl transferase (LCAT) and fecal excretion of bile acids. The deposition of hepatic lipid droplets and hepatic steatosis, induced by the high cholesterol diet, was markedly inhibited by high molecular weight tannins. The cholesterol-rich diet induced oxidative stress in rats, but the high molecular weight tannins significantly increased the activity of superoxide dismutase and glutathione peroxidase (GSH-Px), increasing the total antioxidant capacity, and decreasing the malondialdehyde in serum and liver [114].

*Citrus unshiu* peel (*Citrus unshiu* S. Marcov.) and *Diospyros kaki* fruit (*Diospyros kaki* Thunb.) are renowned traditional herbal medicines, used regularly for treating obesity. The peel of *Citrus unshiu*, is a seedless, easy to peel citrus fruit that has been in use in traditional herbal medicine in Korea and in much of East Asia [112,129].

The prospective anti-obesity agent for decreasing fat absorption by inhibiting pancreatic lipase of unripe *Diospyros kaki* fruits (*Diospyros kaki* Thunb.) and *Citrus unshiu* peel mixture extract (PCM) was studied in vivo and in vitro. PCM is an herbal formulation of *Citrus unshiu* peel (*Citrus unshiu* S. Marcov.) and *Diospyros kaki* fruit (*Diospyros kaki* Thunb.). Its anti-obesity impacts were analyzed centered on the serum lipid parameters assessment from high-fat diet (HFD)-fed mice in vivo. PCM was administered orally at a dose of 50 and 200 mg/kg body weight for a duration of six weeks. PCM demonstrated a repressive impact on lipase activity with an IC_50_ value of 507.01 μg/mL. Additionally, total cholesterol levels, serum triacylglycerol, and visceral fat weight were considerably lowered when related to HFD control mice in PCM 200 mg/kg-treated mice. The total phenolic content was 29.90 ± 0.14 mg GAE/g of PCM extract. The total flavonoid content was 18.33 ± 0.08 mg naringin equivalent (NE)/g of PCM extract. The authors suggested that the administration of PCM could be a different prospective anti-obesity agent for decreasing fat absorption via inhibition of pancreatic lipase [130]. Although the authors did not present the chromatographic profile of phenolic composition of this mixture (just TPC and TFC), this activity was associated with the phytochemistry profile already known for both fruits, and with related scientific works cited by the authors. In the case of *Diospyros kaki* fruit tannins, minerals, carotenoids, vitamins, flavonoids, and dietary fibers [31,93], tannins were mentioned to wield antihypertensive, antitumor, anti-inflammatory, antioxidant, and antidiabetic properties [117,131]. In the case of *Citrus unshiu* peel, hesperidin, naringin, and nobiletin [132] were related to the achievements where these two herbs, *Diospyros kaki* fruit or *Citrus unshiu* peel, played a pharmacological role such as antiadipogenic [133], hypocholesterolemic [134], antioxidant [37], anti-inflammatory [135], and antitumor [136] effects.

The PCM formulation was also tested on non-alcoholic fatty liver disease (NAFLD), an illness that has over the decades become a key cause of chronic liver disease and has been growing around the world in tandem with the astonishing increase in obesity [137]. PCM was orally administered (50 and 100 mg/kg/day) to HFD mice for a duration of eight weeks. Serum TG, ALT, and AST levels in the HFD control mice were substantially greater than the levels found in normal mice. When contrasted with the HFD control mice, supplementing with PCM was found to improve phosphorylation of AMP-activated protein kinase (AMPK). Peroxisome proliferator-activated receptor alpha (PPAR*α*) was substantially increased by PCM administration. Consistently, PPAR*α* activation substantially increased carnitine palmitoyltransferase 1, a crucial enzyme for the process of fatty acid *β*-oxidation, mitochondrial uncoupling protein 2, and thermogenic controlling genes in PCM-treated mice when a comparison was made with those of HFD control mice. Similarly, PCM constrains lipogenesis and constrains cholesterol synthesis through the repression of sterol regulatory element binding protein-1, sterol regulatory element binding protein-2, and its focus genes like acetyl-CoA carboxylase, stearoyl-CoA desaturase-1, fatty acid synthase, and 3-hydroxy-3-methylglutaryl coenzyme A reductase. Altogether, these impacts were mediated through AMPK activation. The authors concluded that PCM enhanced liver damage in HFD-fed mice and diminished NAFLD through PPAR*α* activation and the inhibition of the expression of SREBPs through AMPK-dependent pathways [138].

The genetic regulation involved in cholesterol homeostasis by condensed tannins from the persimmon in two cell lines (HepG2 and Caco-2) was elucidated by Ge and colleagues, who found evidence that tannins in the persimmon extensively modulate the expression of genes involved in the absorption and efflux of cholesterol in cell lines, HepG2, and Caco-2 cells [139]. The intestine acts as a gatekeeper for cholesterol absorption, and the inhibition of cholesterol absorption contributes to the reduction in cholesterol levels in the plasma. Tannins from the persimmon can successfully decrease cellular cholesterol levels in both Caco-2 and HepG2 cells. They inhibited the cell accumulation of cholesterol by reducing the cholesterol biosynthesis-related gene (SREBP-2), the cholesterol absorption-related gene (NPC1L1), and the genes related to the cholesterol efflux regulator (e.g., ABCA1, ABCG1, CYP7A1), regardless of the LXRα signaling pathway [139].

An astringent persimmon ethanolic extract studied in the HepG2 cell line (human hepatoma cell line) according to Hwang and colleagues was shown to have an impact on antioxidant activity, the suppression of cholesterol, and HMG-CoA reductase activity as well as the related molecular mechanism for HDL and LDL. This work also showed that the extract increased LDL receptors and SREBP-2, which controls the LDL receptor, enhances the expression of ABCA1, a cholesterol transporter, lowering LDL and increasing HDL. These results demonstrated that persimmons have antioxidant and hypercholesterolemia preventive effects and can contribute to inhibiting metabolic syndrome [140].

Gato and colleagues also extracted from young persimmons what they called fiber rich in tannins, in order to prepare bars to be included in the daily diet of 40 people, before meals, three times a day, for 12 weeks. It was observed that there was a significant reduction in the levels of total cholesterol and LDL, without changing the levels of HDL or triglycerides in plasma [134]. The tannins of young persimmons include highly polymerized tannins, composed of epicatechin, epigallocatechin, epicatechin-3-*O*-gallate, and epigallocatechin-3-*O*-gallate [134]. Thus, the tannin-rich fibers of young persimmons can be a useful product to prevent and treat hypercholesterolemia, since their daily intake reduced plasma cholesterol levels in humans [134]. Plasma levels of HbA1c were also significantly reduced as a result of the consumption of bars with high doses of fibers rich in tannins, which was related by Gato and colleagues with the capacity of tannins to bind bile acids. As such, fibers rich in tannins can be beneficial in preventing and/or treating type 2 diabetes [134].

Matsumoto and colleagues tested in vitro and in vivo highly polymerized tannins prepared from young persimmons and proved their ability to bind to bile acids, promoting their fecal excretion in mice with 1% (*w/w*) dietary supplementation in tannins, which led the authors to suggest that the polymerized tannins of persimmons are a beneficial food product for the prevention and improvement of the metabolic syndrome [93].

There was also a review on the health effects of proanthocyanidins that indicated that these have potentially beneficial effects in several chronic diseases. They act on cardiovascular diseases, decrease vasoconstriction and oxidation of LDL; in diabetes, the expression of receptors for the final products of advanced glycosylation decreases, lipid peroxidation in the liver decreases; they decrease hyperglycemia, hyperlipidemia, and oxidative stress, causes weight loss, improves insulin resistance, improves nephropathies, retinopathies and neuropathies; in cancer apoptosis increases and proliferation decreases, and angiogenesis decreases; and in bacterial infections, they improve anti-adhesive activity and decrease inflammatory mediators [141].

#### 1.2.2. Proanthocyanidins and Gut Microbiota Modulation

Proanthocyanidins are phenolic compounds with polymeric structures that can be absorbed, since trimeric forms have been detected in rat serum [142]; they may undergo methylation, glucuronidation, and sulfation reactions [143]; or they may not be absorbed and exert local effects on the gastrointestinal tract by neutralizing oxidizing and carcinogenic compounds or be metabolized by colon microorganisms to produce hydroxylated carboxylic acids [144]. Both A- and B-types can be metabolized by the gut microbiota. Metabolites include 2-phenylacetic acid, benzoic acid, 3-(3′-hydroxyphenyl) propionic acid, 2-(3′-hydroxypenyl) acetic acid, 3-phenylpropionic acid, 2-(4′-hydroxyphenyl) acetic acid, and hydroxyphenylvaleric acid. Additionally, 5-(3-hydroxyphenyl)-γ-valerolactone and 5-(3,4-dihydroxyphenyl)-γ-valerolactone metabolites have been found in humans [145,146]. The gut-extracted microbial metabolites of proanthocyanidins are the main transmitting forms in the blood [147]. Their time within human circulation is considerably lengthier than with liver metabolites obtained directly from plant phenolic compounds [148]. Gut uptake and microbiota differences may have an impact on their health effects and on metabolism [149].The limited types of recognized human gut bacteria capable of catabolizing proanthocyanidins and their interactions with proanthocyanidins particularly require further investigation [150,151]. This matter requires that it be tackled soon, in order to comprehend the importance of these compounds [152]. Therefore, they may have a critical role in the prevention of chronic diseases [153]. These include anti-cancer, anti-neurodegenerative, anti-oxidant, anti-osteoarthritic, anti-microbial, anti-diabetic, anti-obesity, cardio-, and eye-protective properties [149].

Proanthocyanidins (360 mg PACs/kg body weight, using male mice C57BL/6J model) promoted the intestinal bloom of *Akkermansia muciniphila*, with the bloom rate being dependent on initial intestinal bacterial abundance and independent of specific intestinal gene expression changes [154]. *Akkermansia muciniphila* is a symbiont that may induce innate immune signaling (via TLR2), supports mucus production, and is an abundant constituent of microbiota in health but not in T1D or IBD [155,156]. In non-obese diabetic (NOD) mice, *A. muciniphila* orally administered induced mucus production and expression of Reg3γ in colon, caused microbiota remodeling, decreased serum endotoxin levels and islet Toll-like receptor expression, and encouraged Foxp3+ regulatory T cells in islets and IL-10 and transforming growth factor beta (TGFβ) in pancreatic lymph nodes [157]. *A. muciniphila* administration to humans appears to be safe, so it is a potential candidate for clinical trials aimed at preventing T1D. Furthermore, it could more easily promote their growth using diet or dietetic supplements as modulatory instruments to induce the *A. muciniphila* growth as an alternative and profitable way to achieve this.

As was demonstrated by the work of Kitabatake and colleagues, dietary supplementation of BALB/c female mice (7-week-old) of a DSS-induced colitis model with tannin extracted from immature persimmon fruits significantly decreased the pathogenesis of ulcerative colitis through the suppression of the inflammatory response and the alteration of the microbiota composition, especially through the suppression of *Enterobacteriaceae* expansion. Gene expression of pro-inflammatory cytokines (TNF-α, IL-6, and IL-1β) and iNOS induced by CpG (short single-stranded synthetic DNA molecules containing unmethylated CpG motifs derived from bacterial DNA) stimulation was meaningfully repressed by the treatment of macrophages (bone marrow-derived macrophages) with the hydrolysate of tannin in a dose-dependent manner. Regarding intestinal microbiota, the relative wealth of Bacteroides was enhanced considerably by tannin supplementation. Microbiota diversity in colitis-induced mice, which were fed tannins, was considerably greater when compared with the control diet group. Furthermore, expansion of *Enterococcus* and *Enterobacteriaceae*, linked with IBD development, was surprisingly inhibited in the tannin diet group. Once again, it highlights that a decidedly safe product obtained from a fruit could become an important candidate for IBD therapy [123].

As a last aspect in this regard, PACs act as hypoglycemic agents, inhibiting COX-2 and IL-1β gene expression. Lycopene, which is a carotenoid, can decrease ROS and grant antioxidant properties the same way as proanthocyanidin [158]. As such, proanthocyanidin should not be considered a miraculous compound, but rather one of the numerous plant secondary metabolites with advantageous bioactivities [159]. 

Adequate valorization of these byproducts presents us with quite a challenge. Ensuring overall system sustainability and reintroduce these byproducts into the food chain will not be an easy task. One example of this effort is the study of blueberry (*Vaccinium corymbosum* L. cv. Duke) and persimmon (*Diospyros kaki* L. var. Rojo Brillante) residues by Bas-Bellver and colleagues, due to their richness in carotenoids and phenolic compounds, to attain powders with elevated added value to be applied as ingredients in food formulation. The characterization of powders and the variations in the bioactive compounds in in vitro gastrointestinal digestion were assessed. Characterization results and in vitro gastrointestinal digestion demonstrated that the drying process, the type of residue as well as the type and content of fiber affects the release of antioxidants throughout digestion. This work indicates that a reduced content of fiber, mainly insoluble fiber (which is what occurred with persimmon powders), could permit for the antioxidant elements to be more available. This was realized from the bioaccessibility index. Then again, bioaccessibility was generally greater for fine powders, however, this was not apparent for blueberry as was concluded by the authors. In vitro colonic fermentations demonstrated that the attributes of digested powders influenced the structure of the developing microbial community. *Streptococcus* and *Veillonella* were substantially more plentiful in persimmon than in blueberry fermentation with high discriminant values. *Odoribacter* and *Butyricimonas* also displayed greater abundance in persimmon than in blueberry fermentation. In contrast, the Firmicutes genera, *Ruminococcaceae* GCA_900066225, *Pygmaiobacter*, and *Lactobacillus* demonstrated considerably greater richness in blueberry fermentation. The authors attributed the *Streptococcus* as a biomarker of persimmon fermentation, possibly because the high content of anthocyanins and fiber affect *Streptococcus* growth. Moreover, separate studies have demonstrated the selective bacteriostatic or bactericide effects of phenolic compounds on fecal microbiota, primarily potential pathogen [160]. On the other hand, phenolic compounds such as anthocyanins are able to stimulate the development of helpful bacteria *Akkermansia muciniphila*, *Bifidobacterium*, *Lactobacillus,* and *Faecalibacterium prausnitzii* [161]. An elevated degree of fiber and anthocyanins has a harmful effect on *Streptococcus*, but stimulates *Ruminococcaceae* genus and *Lactobacillus* growth. Encouraging associations were also identified between phenolic compounds and *Actinobacteria genera* (*Collinsella* and *Bifidobacterium*), *Akkermansia*, and *Ruminococcaceae_UCG*−014. The content of fiber is positively associated with *Faecalibacterium* and *Bifidobacterium*. In light of these results, the authors suggest that blueberry bagasse and persimmon waste powders might be incorporated in the food formulation to enhance anthocyanin and carotenoid content, which might have an impact on human health [162].

Based on the results obtained, Lee and colleagues suggested that the antioxidant effect of the persimmon proanthocyanidins can be estimated for a consumption of around 50 g/day in the case of adults with a body weight of 60 kg [163].

### 1.3. New Products and Byproducts Valorization

What kind of diet can our planet reasonably support in order to feed 2050’s estimated population of 10 billion human beings? To answer this question, one needs to draw from areas such as agriculture, nutrition, and climate research [24]. When thinking about a sustainable food system revolution, there are five research and action areas that need more consideration: political economy, social justice and equity, structural and economic costs, tools to support governance and decision, and cultural norm diversity [164]. For many countries, it is inexpensive to improve diets, however, for many around the planet, this leap would necessitate a recipe combining lower prices, nutrition assistance, and higher incomes. Data and analysis on the cost of healthier foods is essential in order to properly advise both local interventions and systemic changes [23].

#### 1.3.1. Reformulation of Traditional Foods (Spaghetti; Pork Liver Pâté; Rice Noodles; Cheese; Yogurt; Cupcakes; Persimmon Pulp; Hot-Air-Dried Chips; Ale Beers; Vinegar; Probiotic Food Products; Emulsifier)

The globalization of fruit and vegetable markets has led to overproduction, surpluses, and the generation of potentially valuable residues. The global urgency for measures to protect the environment places great pressure not only on how food is produced, but also on how the waste produced may be diminished. Valuing waste residues by discovering new food ingredients, new foods, or even new supplements, waste can be reduced. Science has, for some years now, brought valuable knowledge on how we may develop systems of sustainable nutrition. 

While there is yet much to discover, it is urgent that there be a valorization of data already known for applications that can impact food markets, bringing new ways to promote population health, while decreasing the environmental impacts that agriculture and the food industry are known to have on the planet.

Even agricultural waste such as persimmon peels are a good source of proanthocyanidins and the extraction of these compounds from the refuse of abundant natural resources can be a sustainable way for dietary supplement and functional food development [159].

Lucas-González and colleagues have previously demonstrated that persimmon flours (cultivars ‘Rojo Brillante’ and ‘Triumph’) from juice coproducts could be a source of bioactive compounds. During in vitro digestion, the recovery index for flavonoid and phenolic content was dependent on the type of flour and the phase of digestion. The antioxidant activity [2,2′-azino-bis (3-ethylbenzothiazoline-6-sulfonic acid) radical (ABTS^•+^), ferric reducing antioxidant property (FRAP) and 2,2-diphenyl, 1-picryl hydrazyl (DPPH)] decreased after the intestinal phase, while their chelating activity (ferrous ion chelating assay) increased in both flours. Given all that the authors suggested, persimmon flours might (or rather should) be incorporated in food formulation to enhance either the scarcity of bioactive compounds or an unbalanced nutritional composition [165].

An example of this is persimmon flours (Triumph and Rojo Brillante) at low concentrations that were employed in the development of spaghetti with higher polyphenol content, which exhibited lower starch digestibility than that of traditional spaghetti. The work of Lucas-González and colleagues demonstrated that the in vitro gastrointestinal digestion of durum wheat semolina spaghetti enriched with two types of persimmon flour (“Rojo Brillante” flour and “Triumph” flour) at two concentrations (3 and 6%) increased the total phenolic compound content in a dose-dependent way and apports gallic acid and coumaric acid-o-hexoside, which were not present in the control spaghetti. After in vitro digestion, a high number of phenolic compounds continued to link to cell wall or indigestible polysaccharides and as such, could be used by gut microbiota. The 3% persimmon flour-enriched spaghetti decreased the digestion of kinetic starch, while 6% enriched spaghetti increased it. However, the predicted glycemic index was similar between the control and enriched spaghetti. Spaghetti with 3% persimmon flours showed a glycemic index lower than spaghetti with 6% persimmon flours. With these results, the authors concluded that persimmon flours at low intensities may well be applied in the development of spaghetti with increased polyphenol content and less starch digestibility than traditional spaghetti [166].

Reformulating traditional foods to cut synthetic additives being used or improving their end-product quality by making use of co-products from the agro-food industry is a path that may be a factor toward improving the chance for a second green revolution.

Pork liver pâté, for instance, is generally eaten throughout Europe, which delivers vitamins, protein, and fat. However, given its high-fat content and low natural antioxidants, it suffers from lipid oxidations [167], which has adverse implications for health and as such, presently, there is an increasing demand for natural ingredients. Agro-food co-products might serve as a resource of natural ingredients with distinct technological properties such as colorants, antioxidants, emulsifier agents, or water bindings. Additionally, certain characteristics have elevated quantities of nutrients such as minerals, vitamins, fiber, and phytochemicals [168,169].

Possibilities have been studied such as the reformulation of pork pâté with persimmon flours. Enriching this kind of pâté with persimmon flours triggered a decrease in their total cholesterol content and lipid oxidation after in vitro digestion, with no alterations in their fatty acid profile as to what the phenolic compounds could be influencing. This work resulted in the cholesterol content in pâté samples being considerably decreased in a dose-dependent way (control > pâté 3% > pâté 6%; 98 ± 8; 89 ± 3; 68 ± 11 mg/100 g pâté, respectively). The caffeic acid, gallic acid, the aglycone and glycosylated forms, glycosylated quercetin, and glycosylated coumaric acid were detected in the supplemented pâtés. These compounds in total were 74 and 239 µg/g pâté in the pâtés with respectively 3% and 6% of persimmon flour. Linoleic, palmitic, and oleic acids represented most fatty acids found in all pâtés. The rise of lipid oxidation after in vitro digestion was greater in the control pâté than in the supplemented pâtés [170]. These types of foods, which are rich and fatty, are exceptional in safeguarding bound phenolic compounds, and may get to the colon unharmed where they may then be metabolized by the intestinal microbiome. Lucas-González and colleagues prepared the following: a control pâté without the addition of any persimmon flour (CP); an enriched pâté with 3% persimmon flour obtained from ‘Rojo Brillante’ persimmon juice coproducts (PR-3); and an enriched pâté with 6% persimmon flour (PR-6). This research team detected within the persimmon flour 42 polyphenolic compounds, 37 of which were identified, and 12 were subsequently confirmed by standards (gallic acid, catechin, caffeic acid, epigallocatechin-3-gallate, gallocatechin-3-gallate, *p*-coumaric acid, epicatechin-3-gallate, ellagic acid, ferulic acid, myricetin, quercetin, and kaempferol). Persimmon flour might be deemed to be a decent source of insoluble bound phenolic compounds, particularly gallic acid, to augment meat products. Nevertheless, PUFA stability is adversely affected, inducing their oxidation, particularly when combined at the highest concentrations (6%). Using two pancreatins that had distinct lipase activity (8 UL/mL and 2000 UL/mL) significantly impacted both lipid oxidation and the stability of bound phenolic compounds. The maximum amount of bound phenolic compounds and TBARs quantities was reached after the intestinal phase C2, where the greater the frequency of lipolysis, the greater the number of fatty acids in the environment, which induced protection of phenolic compounds against degradation and lipid oxidation. Between digested and undigested pâté samples, low variations were shown among the fatty acid profiles. Remarkably, their contents of PUFAs rose after both intestinal phases, the reason most likely being that this digestion phase enhanced their extractability. Lipid oxidation was decreased in pâtés in a dose-dependent way by persimmon flour after both C1 and C2 intestinal phases. Even though the R-6 pâtés demonstrated greater oxidation than that of the control, it was not improved after digestion. Thus, it may be determined that lipase activity is a critical aspect that needs to be considered in the intestinal phase of the in vitro digestion activity. Nonetheless, further research, both in vitro and in vivo, is required, not to mention the contribution of further knowledge on in vitro bile holding ability, colonic fermentation, lipid digestibility, and phenolic compound transformations with the purpose of achieving a more comprehensive view of the health implications that might be beneficial in reinforcing the fitness of meat product amelioration with persimmon flour coproducts [171].

Another example of the reformulation of traditional foods becoming healthier with the addition of vegetal byproducts and becoming more attractive to consumers who are increasingly ecofriendly, are rice noodles. Immature persimmon juice for sensory and color properties of rice noodles have been explored in the pasting and textural sense. Adding persimmon juice caused a considerable rise in peak viscosity, rapid visco-analyzer setback, and final viscosity, however, there was a reduction in the pasting temperature of rice flour. The addition of immature persimmon juice to rice flour may well improve the textural properties of rice noodles and impart color stability. Microstructural examination from both laser scanning confocal microscopy and scanning electron microscopy revealed that cooked juice-containing noodles had a more homogeneous and compact microstructure. Results of this work provided the theoretical foundation for the use of wasted fruit products and a practical guide to produce rice noodles with improved nutrition and sensory quality. The authors of this study back in 2012 postulated that immature persimmon juice could be suitable to add to rice noodle formulation [172].

Research that assessed the impact of persimmons on the characteristics of sweet or salted-processed cheese was conducted to create a new type of cheese with elevated nutritive value. In addition to the control sample, a preparation of two types of kaki cheese was undertaken: the first was conducted by merging 20% kaki juice and 12% sugar, while the second by adding 20% kaki juice and 5% table salt. Salted and sweetened kaki processed cheese-spread samples were prepared by making use of a cheese base. The organoleptic assessment demonstrated that the fresh cheese samples with 20% kaki juice and 12% sugar achieved the highest scores for flavor, appearance, and overall acceptability. Nevertheless, the assessment also demonstrated that it had low degrees in texture, color, and spreading quality. The prepared processed-cheese-spread samples using kaki fruit were available with acceptable properties [173].

Yogurt is another product that has been examined using persimmon fruit. The increase in functional and nutritional characteristics of yogurt by combining it with persimmon and evaluating a valuable and suitable use of persimmon fruit in yogurt production in various concentrations (10% and 12%, *w/w*) of persimmon marmalade and puree used in yogurt production. The yogurt that contained the 12% persimmon marmalade obtained the top sensory evaluation values. The greatest viscosity and water capacity rates were identified in the 12% persimmon marmalade yogurt sample on the 15th day. Samples made by combining them with persimmon puree exhibited lower antioxidant activity than the other samples. In the production of yogurt, the use of persimmon marmalade provoked a rise in acidic taste, appearance, structure, odor, taste, perceived fruit taste, perceived sweetness, and overall acceptability scores. The use of dried persimmon in the puree’s production might have been the cause for the low sensory scores attained with yogurts containing persimmon puree. Counts of the samples of *Lactobacillus delbrueckii subsp*. *Bulgaricus* and *Streptococcus thermophilus* diminished during the storage period. Evaluators preferred the persimmon marmalade yogurt samples. The impact of adding different persimmon forms on the structure, appearance, taste, odor, acidic taste, perceived fruit taste, perceived sweetness, and overall acceptability was considerable. This study thus concluded that persimmon marmalade may be used in yogurt production successfully, which is also an alternative use for persimmon, which has a short shelf life [174].

Cupcakes were prepared by adding differing amounts of persimmon puree (33.3, 50, 66.6, and 83.3%) in order to improve the nutritional and antioxidant activity of cupcakes. The work carried out by Abdallah and colleagues studied the physical, chemical, and bioactive compound characterization of persimmon puree and evaluated the effect of employing persimmon puree on some characteristic factors of cupcakes. The data demonstrated that using persimmon puree in the production of cupcakes at a ratio of 33.3%, may be capable of improving the physical, chemical, and organoleptic traits as well as cupcake antioxidant activity. The persimmon puree cupcake had larger amounts of vitamin C, total phenolic compounds, and antioxidant activity than those found in the control sample. Furthermore, cupcakes with persimmon puree had superior scores for organoleptic properties than those found in the control sample [175].

As already mentioned, persimmon fruit is a highly perishable food product that spoils quickly, so it needs to be converted into edible products. An important way to use fruits and vegetables while avoiding waste is to produce semi-finished products such as juices, purees, pulps, etc. The contribution of science to this issue comes with work such as the one presented by Gautam and colleagues. In it, the authors tested several possibilities for the optimization of pulp preparation from ripe persimmon (fully ripened persimmon fruits of Fuyu variety) to assess the best preservation method for storage. From nine treatments using fruit with peel and nine treatments using fruit without the peel that were tested, fruit with peel was selected based on nutritional and sensory features and the treatment of pulp with pasteurization and with 1000 ppm potassium metabisulfite (KMS) in glass bottles was identified as the best type of pulp. The product was kept at ambient temperature and quality was evaluated at 0-, 3-, and 6-month storage periods. This treatment maintained the highest amount of β-carotene (173 and 86 µg/100 g), ascorbic acid (13.733 and 8.043 mg/100 g), and total phenols (3.093 and 2.873 mg/100 g), respectively during storage for six months. This study recommended that nutritionally rich persimmon pulp may be formulated with better storage stability, which can be employed by both small- and large-scale industries at a lower manufacturing cost [176]. Other methods and variations could be discussed and tested, and more knowledge could be added to this issue, which will certainly increase the persimmon’s market value and crop valorization, reducing the waste and making its production and scale-up profitable.

The development of hot-air-dried chips as a product with added value for persimmons has been taking place recently and is another option to optimize the valorization of this fruit and its production. The processing conditions and two packaging types were explored by Milczarek and co-workers. Fresh persimmon fruits (cultivar “Hachiya”) were used, and the dried chips were prepared in the following manner: preparations 2–5 had the fruit sliced at a thickness of 2 mm, which was then hot-air-dried for a duration of 5 h; and preparations 6–10 had the fruit sliced at 6 mm and subsequently dried for 10 h. These preparations were then packaged into two types of bags, either a plastic zip-top bag or a metallized polyethylene terephthalate (Met-PET) bag, which were then stowed in ambient settings, and subsequently sampled for moisture-related, texture, microbial, and chemical quality characteristics all the way through the 1-year storage study. Chemical analyses included phenolic compounds, antioxidant activity, and tannins content dosage. Additionally, the instrumental texture assessment of the dried persimmon chips, microbial assessments, color, water activity, and moisture content (wet basis) of the samples were tested. It was discovered that apart from a clear decline in ascorbic acid, all four preparation and packaging combinations generated products that retained adequate nutritional, microbial, color, and texture quality all the way through the whole study. It is suggested that hot-air-drying persimmon slices is an encouraging method to create a product with added-value that has a minimum of a one-year shelf-life [177]. Partial least-squares regression (PLS) was employed to create models that predicted the sensory characteristics from instrumental measurements, and models that predicted the consumer liking score from both instrumental measurements and sensory attributes. These PLS models identified the texture characteristics that the consumers wanted the most for this product, and the preferences were clear: consumers preferred a smooth and moist texture rather than a more typical crispy and dry texture, which was ironically the texture that was originally intended [178]. With these studies, a new type of food produced 100% from persimmon emerges as an option for the consumer.

In order to raise its accessibility to consumers and increase persimmon’s (*Diospyros kaki* L.) value, the creation of special beers (fruit ales) with distinct concentrations (100:0%, 75:25%, 50:50%, and 25:75% *v/v*) of persimmon juice (‘Rojo Brillante’ variety) and barley malt were analyzed by Martínez and colleagues. Fermentation was then undertaken in strict beer quality-control parameters, and the persimmon juice’s influence on quality was explored. Color, titratable acidity, total PCs, sugars, total soluble solids, turbidity, pH, organic acids, antioxidant capacity, and ethanol formation were all verified throughout the process of fermentation. These ales, whose alcoholic contents were well within the standards (3.6–5.63% *v/v* ethanol), were categorized as having a normal acid pH (between 3.97–4.13) with lactic and citric acids as the most abundant organic acids, a clear golden color without turbidity (between 2.05–2.83 in European Brewery Convention units), intermediate total phenolic compound values (283.0–327.1 mg GAE/L), and antioxidant activities between 1.65 and 5.78 mM Trolox equivalents/L. The beer samples 50:50% and 25:75% wort–persimmon juice showed constant TPC values during all fermentation process. The result was a beer with a fruity aroma whose quality parameters were within the standards. Overall results showed that production of both persimmon beverages (25:75% wort–persimmon juice) and 50:50% wort–persimmon beer might open new uses for persimmon fruits in the food industry. The persimmon beverage that was the most appraised and favored by the evaluators was the one that contained 75% fruit juice, with the 50:50% wort–persimmon beer being the beverage that was preferred second [179]. One of the most famed beverages in the world has been reinvented in the last decade, with new recipes, new components, new proportions, and combinations of ingredients. Fruits, for instance, are added for flavoring beer, where adding fruit to wheat is commonplace given that it contributes toward texture upgrade. The influence that persimmons have on antioxidant activities as well as the quality characteristics of beer have been tested by scientists over the last decade. In the study performed by Cho and colleagues, antioxidant activities determined through DPPH and superoxide and hydroxyl anion scavenging potentials as well as total PCs of the persimmon-enhanced beer were substantially superior in comparison with the control. The beverage’s mineral profile, which contained Ca, K, and Mg, was also considerably higher and there were no toxic heavy metals detected in the persimmon beer. The general acceptance value was substantially elevated when the beer was prepared by adding 150 g of the fruit to 10 L of water. Results indicated that this addition of 150 g of persimmon per 10 L of water could more successfully enhance the antioxidant, organoleptic, and nutritional capabilities of beer [180].

In addition, vinegar is a cheap product due to its inexpensive production from its raw material. A new alternative source for vinegar production is persimmon processing byproducts such as persimmon peels, which are generally dropped during the consumption or processing of this fruit. The persimmon peel vinegar tested by Bayram and colleagues demonstrated a good antimicrobial effect in agar diffusion method against five bacteria (*Escherichia coli* O157: H7 ATCC 33150, *Bacillus cereus* FMC19, *Listeria monocytogenes* ATCC 19118, *Staphylococcus aureus* ATCC 25923, *Salmonella Typhimurium* ATCC 14028) and three molds (*Aspergillus flavus, Penicillium carneum,* and *Aspergillus niger*). Generally, upon increasing the extract concentration, the inhibition zone was also increased, and the highest inhibition zone was observed at direct (100%) extract concentration applied against *Escherichia coli* for antibacterial activity and the highest inhibition zone was observed at direct (100%) extract concentration applied against *Aspergillus niger* for antifungal activity. The described vinegar had health-promoting qualities and might be a competitive product in the commercial market. Through fermentation, persimmon vinegar can be produced and it is projected as having functions physiologically, given that it is abundant in potentially bioactive compounds of the persimmon fruit, not to mention as valuable substances generated by microorganisms throughout the process of fermentation [181]. A traditional Korean food that is fabricated and consumed by ordinary farm households is persimmon-vinegar, where it is utilized as a folk remedy, typically for overcoming fatigue or managing hangovers. Another study demonstrated that vinegar made from persimmon reduced the deposition of fat and ameliorated hepatic function in mice administered orally with alcohol [182]. The importance of this production increases even more if it is possible to guarantee a sustainable production, which becomes relevant if we keep in mind the health benefits already scientifically demonstrated. This is the case with the study of polysaccharides isolated from Korean persimmon vinegar, which had the most potent macrophage-stimulating activity among the six tested vinegars from other raw materials (Korean brown rice vinegar, Korean apple vinegar, Korean persimmon vinegar, wine vinegar from the United Kingdom, Korean wine vinegar, Japanese brown rice vinegar) [183].

Polysaccharides are a class of additives that are widely employed in the drug and food industries. They have a vast therapeutic property spectrum, comparatively low toxicities, not many side-effects, and they possess distinctive physical, chemical, and biological characteristics, which has led to them being extensively explored [184]. Polysaccharide effects (immunomodulatory, antitumor, and anti-inflammatory) have been attracting increased interest within the biomedical fields [185]. Isolated crude polysaccharide taken from Korean persimmon-vinegar tested on intestinal immunostimulation demonstrated no cytotoxicity to intestinal epithelial Caco-2 cells, also demonstrating that it could be transported through a Caco-2 cell monolayer in an in vitro coculture system. In the in vitro experiment, the Korean persimmon-vinegar treatment considerably improved the production of IgA by Peyer’s patch cells and prompted an upsurge in transforming growth factor (TGF)-β1 and interleukin (IL)-6 values. The in vivo impacts of the Korean persimmon-vinegar study on mice that were orally administered at different doses for 20 days showed that the oral administration of this vinegar induced IgA and cytokines (IL-6, granulocyte macrophage-colony-stimulating factor, and TGF-β) production by Peyer’s patch cells and significantly increased IgA levels in feces and intestinal fluids. The findings of this research imply that the persimmon-vinegar isolated polysaccharides seem to modulate the intestinal immune system and might be advantageous to human health [186].

Persimmon-vinegar is present in probiotic food products such as kimchi and cheonggukjang (a Korean traditional fermented soybean product), which are traditional Korean foods, commercialized as Mogut^®^. The cheonggukjang and kimchi probiotic ingredients are as follows: a culture medium with fermented bacilli of kimchi and cheonggukjang (*Leuconostoc mesenteroides, Leuconostoc holzapfelii,* and *Lactobacillus sakei*; 99.7%), Hasuo extract (*Pleuropterus multiflorus*; 0.1%), persimmon-vinegar (0.1%), and Korean black soybean extract (*Rhynchosia volubilis Lour*; 0.1%). In a study aimed at assessing whether probiotics could benefit patients suffering with alopecia, forty-six patients were administered with 80 mL of Mogut^®^ (Mogut^®^, Seongnam, Korea), two times a day before breakfast and before bedtime. Parameters related to hair were evaluated after one month and four months of administration and were then compared with the baseline (month 0) values. In all patients (n = 46), the baseline hair count was 85.98 ± 20.54 hairs/cm^2^ and the thickness was 0.062 ± 0.011 mm. The hair count and thickness had substantially improved one month after the treatment began (90.28 ± 16.13 hairs/cm^2^ and 0.068 ± 0.008 mm, respectively) and at four months (91.54 ± 16.29 hairs/cm^2^ and 0.066 ± 0.009 mm, respectively) when compared to the baseline value. A total of 93% of all patients exhibited positive impacts in terms of the hair parameters that were evaluated (thickness and hair count). The effectiveness of the cheonggukjang and kimchi probiotic product was different for men and women. The results showed that it was more likely for men to have an impact only with hair count (26.1%) or only with hair thickness (30.4%), as opposed to an impact on both parameters (39.1%). In comparison, most women demonstrated a beneficial impact on both hair parameters that were assessed (65.2%), and only 13.0% and 8.7% of women showed corresponding improvements in either only hair thickness or only hair count. The researchers of this work noted that the administration of the cheonggukjang and kimchi probiotic product was ineffective in 8.7% of women and 4.3% of men [187].

Probiotics could have beneficial effects that extend beyond gastrointestinal health, as they have demonstrated efficacy in improving certain metabolic disorders such as hypertension [188], hypercholesterolemia [189], and atherosclerosis [190]. Metabolic diseases like hypercholesterolemia can have a harmful impact on microvascular function, which can be reversed with the help of a cholestyramine lipid-lowering therapy [191]. With a basis on the research performed by Park and colleagues, the consumption of adequate amounts of probiotics might produce a beneficial effect on peripheral vascular blood flow that impacts hair growth. The kimchi and cheonggukjang probiotic product analyzed in this study included different kinds of probiotics (*Leuconostoc holzapfelii, Leuconostoc mesenteroides*, and *Lactobacillus sakei*; 99.7%) and prebiotics obtained from kimchi, cheonggukjang, and natural herbs [187], where persimmon-vinegar was also included.

Persimmon peels can serve as a new source of emulsifier with antioxidant activity, providing a promising alternative for natural emulsifiers that can be used in the food industry. Emulsifiers are essential for the food industry, their common applications mainly consist of improving the texture and taste of food and raising the stability of disperse systems [192]. The recent population demand in the last decade for natural emulsifiers have led to there being more attention being paid due to their food acceptability and plentiful sources [193]. In this demand for natural emulsifiers for food manufacturers, pectin has attracted more and more attention due to its emulsification properties. Therefore, the development of new pectin sources has obvious advantages over enzyme and chemical modification in food acceptability [194]. The structural and functional characteristics of persimmon pectin are still poorly understood, but scientific studies about this subject are in development. To this purpose, Jiang and colleagues explored the molecular weight distribution, thermodynamic characteristics, the physicochemical analysis, FTIR spectrum, NMR spectroscopy (1H), and XRD pattern from pectin extracted (hot-acid extraction) from persimmon peel. Commercial citrus pectin was used as a control to compare the emulsifying properties of persimmon pectin including emulsifying ability, stability, and emulsion microstructure. Emulsification performance showed that when the pectin concentration was increased to 1.5% (wt%), a stable emulsion containing 50% or 60% (*v/v*) oil fraction can be obtained, which had excellent emulsifying properties. These results indicate that the emulsifying properties of persimmon peel pectin may depend to a large extent on the hydrophobic interaction of acetyl groups and the cross-linking of tannins with pectin. The structural characterization indicated that the obtained persimmon peel pectin belongs to a type of low-methoxy acetylated pectin. In addition, persimmon peel pectin’s own superior antioxidant biological activity provides additional lipid oxidation stability to the oil phase of the emulsion. These achievements suggest that persimmon pectin can be used as a new type of food polysaccharide emulsifier and the development of an emulsifier with its own antioxidant activity may be an effective strategy to prevent lipid peroxidation that could help in the development of persimmon pectin-based emulsifier [195].

#### 1.3.2. Antimicrobial Activity and Food Packaging

*Helicobacter pylori* is a flagellated, spiral-shaped, urease producing microbe that infects around 50% of humans around the world [196]. With therapies using antibiotics, a combination of three antibiotics such as clarithromycin, amoxicillin, tetracycline, and proton-pump inhibitors (omeprazole, pantoprazole, etc.) are greatly applied as an effective chemotherapeutic method [197,198,199]. The current anti-*H. pylori* therapy may lead to undesirable side effects, and the high prevalence of antibiotic-resistant strains limits their frequent use during chemotherapy [200]. Furthermore, the enzyme urea amidohydrolase production increases *H. pylori* gastric acid resistance; urease hydrolyzes urea producing ammonia and carbon dioxide, and the end-products then nullify stomach acid to generate a pH environment fit for the establishment and proliferation of *H. pylori* [201]. According to an animal model experiment, *H. pylori*’s urease knockout mutant does not seem to be responsible for causing gastric diseases [202]. As such, urease is thought to be a key focus for repressing *H. pylori* colonization [203]. Additionally, peptide deformylase presents itself in the human body, but in protein synthesis in humans, the enzyme is not involved, but it is, however, involved in protein synthesis in bacteria (*H. pylori*). As such, this enzyme may be an additional potential target for the development of anti-*Helicobacter pylori* drugs [198].

The pedicel of *Diospyros Kaki.* L., which is always a waste from this fruit consumption, could be applied to fight against *Helicobacter pylori.* The work of Saravanakumar and colleagues demonstrated that pedicel extracts have anti-*Helicobacter pylori* agents and the synergism of various compounds in the pedicel extracts increased the anti-*H*. *pylori* effect. Saravanakumar and colleagues performed the analysis of alkaloids and low molecular weight compounds from the pedicel extract and their inhibitory efficiency on *Helicobacter pylori* urease and peptide deformylase. To test the inhibitory effect against growth and pathogenicity related enzymes, urease, and peptide deformylase were used in the systematic virtual screening approach. For this purpose, the dried persimmon pedicels were powdered and exposed to a chloroform and ethyl acetate systematic extraction. The final extract was lyophilized and named as PDK-EAE (*D. kaki* ethyl acetate extract) and PDK-CE (*D. kaki* chloroform extract) before dissolving in DMSO for the bioactivity assay including the antioxidant (DPPH and hydrogen peroxide free radical scavenging activity) and antibacterial assay [204]. Disc diffusion method [205] followed by calculating the minimal inhibitory concentration by using the microdilution method were used in order to evaluate the antibacterial activity of PDK-EAE and PDK-CE, followed by the evaluation of the effect of PDK-CE on bacterial cells (*H. pylori*) by high-resolution transmission electron microscopy (HRTEM) according to Hickey and colleagues [206]. In the HRTEM analysis, the MIC of PDK-CE was treated for 24 h at 37 °C to *H. pylori*, and the urease inhibitory activity of the PDK-EAE and PDK-CE was established through Berthelot reactions [207]. It is also worth mentioning that the systematic virtual screening measured the inhibitory effect determined through binding mode and theoretical affinity of metabolites from PDK-EAE and PDK-CE against urea amidohydrolases and peptide deformylase by a computational molecular docking method [204].

All the pedicel extracts from *D. kaki* proved to be non-toxic and to have H_2_O_2_, DPPH, and NO radical scavenging activity. The molecular interactions that are taking place between traditional antibiotics and peptide deformylase (HPPD) suggested that the traditional antibiotics’ docking energy scores against HPPD were −8.25 kcal/mol for amoxicillin, −7.54 kcal/mol for novobiocin, and −3.59 kcal/mol for cefuroxime. Amid the antibiotics that were examined, amoxicillin demonstrated the greatest docking energy score versus HPPD with the solid binding capacity with hydrophobic and hydrophilic residues (Asn62, Leu59, Leu132, Leu63, Ile45, and Ile60), and its binding pockets contained other hydrophobic and neutral residues (Leu47, Leu76, Leu126, Leu 131, Ile78, Ile114, Ile136, and Ile148). The pedicel extract-derived compound demonstrated the HPPD docking affinity and binding fit. However, among the compounds, the compound that displayed the higher docking energy of −14.51 kcal/mol was hexadecanoic acid with the strong interactions of hydrophobic residues (Ala135, Ile136, Leu59, Leu76, Leu78, Leu132, and Tyr116) while also showing the presence of other residues (Ile114, Ile140, Pro81, Leu 63, Leu126, and Leu131) in its binding pockets. As such, the survey of the molecular docking revealed a synergistic action of pedicel extract derived chemicals on preventing *H. pylori* through the suppression of *H. pylori* urease and HPPD. Based on this information, the authors suggested that *D. kaki* could be a hopeful source for the development of a novel drug against *H. pylori* infections [204] based on natural products and on food waste.

Persimmons could have other roles such as in the preservation of food, as demonstrated for persimmon tannins that exhibited antimicrobial action counter to foodborne methicillin-resistant *Staphylococcus aureus* (MRSA) and may well be developed in theory as a replacement or supplement to manage the propagation and infection of MRSA in foods [208].

At present, some MRSAs have been extracted from a variety of food products (ex. milk, cheese, retail beef, pork [209]), which could be tainted by the handling of humans as well as the infection of farm workers and livestock [210]. Consuming products like these may lead to food poisoning by *Staphylococcus*, which may be triggered by a variety of virulence factors. However, there are some aspects to consider, namely that there is also an increased morbidity and mortality potential, multiple drug-resistance, aggressive course, and hospital outbreaks [211]. A number of anti-MRSA antibiotics have been introduced for medical treatment, some of which are daptomycin, vancomycin, linezolid, teicoplanin, telavancin, and new glycopeptides [212].

If we take into consideration how the antimicrobial resistance of MRSA has become ever-increasing, innovative substances (not counting antibiotics) have gained enormous attention in the search for such new alternatives. Compounds derived from plants such as alkaloids, flavones, polyphenols, and tannins, extracted from medicinal and horticultural plants [213,214], have been used with success to battle numerous bacterial infections, providing a possibly rich source of novel alternatives to supplement diminishing antimicrobial strategies [215] for the food processing industry.

Persimmon tannins from young astringent persimmon fruits actively inhibited several MRSA isolated from retail pork where the minimum inhibitory concentrations (MICs) were 1000 μg/mL. Adding persimmon tannins caused bacterial membrane integrity decrease, serious cell wall and membrane damage, which led to membrane hyperpolarization, a reduction in intracellular ATP concentration, whole cell protein and cycle depression, not to mention the morphological modifications [208]. The results presented by Liu and colleagues suggested that persimmon tannins from astringent young fruit can be used in the food and medical industries to inhibit the growth of MRSA. Further research is required on the large-scale extraction and purification of persimmon tannins as well as their impacts on the organoleptic characteristics of foods before commercial applications are to be considered [208].

In addition, all the innovative postharvest technologies to preserve and extend the shelf life of minimally processed vegetables (MPV) is especially important currently due to the large demand by consumers for fresh foods without chemical additives [216]. In this context, Matheus and colleagues developed a biodegradable and edible film created from persimmon (*Diospyros kaki* L.), which was employed as a cover for MPV packaging. A biodegradable film based on persimmon with glycerol and pectin was developed and the effect of storage time on microbiological and physical properties of minimally processed carrot, beetroot, and cucumber packed in plastic bowls covered by a persimmon film lid was assessed. PVC was used as the positive control. The film with smaller water vapor permeability and thickness, decent flexibility, greater tensile strength, and a more vibrant color was chosen as a lid for MPV bowls. Vegetables that were wrapped with PVC or film produced results that were similar concerning thermotolerant coliform evolution; psychrophiles and fungi count; pH value (cucumber 5.95–6.18; carrot 5.96–6.10; and beetroot 6.02–5.75) as well as optical characteristics. Persimmon film decreased the MPV dehydration process, even if to a lesser extent than that of PVC [217]. The potential that persimmon-based film shows as a strong contender to synthetic covers for vegetable packaging is being actively explored and has thus far presented new and exciting possibilities.

Taking into consideration the work done on the preservation of food products such as postharvest technologies to preserve and extend the shelf life of minimally processed vegetables with biodegradable and edible films based on persimmon (*Diospyros kaki* L.) used as a lid for MPV packaging [217] and the work of Liu and colleagues about anti-microbial activity exhibited by persimmon tannin fruit against several MRSA isolated from retail pork (MIC of 1000 μg/mL) [208], it could be hypothesized that the possibility of applying the technology developed by Matheus and colleagues [217] against several MRSA [208] and applying this to the food processing industry (specifically to meat processing industry) is an interesting prospect. Public health and the health system would have advances combined with environmental gains from the use of biodegradable packaging with edible film based on persimmon or persimmon byproducts.

#### 1.3.3. Fabric Dyes

In a related aspect, dyes are ubiquitous in various areas of application ranging from the cosmetic, tannery, textile, and food industries to veterinary and human medicine. Their widespread applications and large-scale production have caused synthetic organic dyes to seep into the soil and water. Dyes are taken too lightly when seen as aquatic environment pollutants. The presence of dyes in aquatic environments has dire implications for various ecosystems, but extraordinarily little is known regarding synthetic organic dyes as contaminants of water bodies. The presence of dyes in water bodies may very well be a reason for aquatic animal contamination, however, thus far, dyes have been established in environmental samples such as water, sediment, suspended particulate matters, and wild fish [218].

In this context and from the perspective of an eco-friendly alternative reductant, persimmon peels could also be useful. An extract of persimmon peel may be applied for indigo reduction dyeing as a nonhazardous, sustainable, eco-friendly alternative to sodium dithionite. Yoo and colleagues developed an extract from persimmon peels (from non-astringent persimmon) where they detected respectable antimicrobial activity against *Staphylococcus aureus*, and tetracycline was used as a positive control. Persimmon peel extract inhibition areas were produced 2 mm at 5 mg/disc and 4 mm at 10 mg/disc. This feature was related by the authors of this study as able to assist in keeping the reduction bath from ruining or deteriorating. The greater the extract concentration, the greater the color strength and the longer thee reduction time that was maintained. It is thus likely that the reduction baths may be utilized frequently by supplementing the extract powder. Indigo bath with persimmon peel extract redox potentials were similar to the results produced in the sodium dithionite reduction bath. Even though the sodium dithionite reduction bath led to higher ramie fabric color strength, the reduction state was preserved for a shorter amount of time, around 2–3 days, in comparison with the persimmon peel extract that was preserved for more than 10 days [219].

In the field of fabric production, persimmon juice was also tested with the purpose of dyeing silk and cotton fabrics. The treatment of silk fabrics with persimmon juice through padding demonstrated that dyed fabrics subjected to UV light ended up having a more intense yellow-red color than that of those exposed to sunlight. Silk fabrics treated with premordants exhibited strong yellow colors, especially those fabrics where the Fe mordant was added showed greenish red-yellow colors. Given that the use of persimmon juice increases the padding times of dyeing, properties such as strength and water repellency are improved along the corresponding warp and weft directions, although the anti-crease property was shown to be reduced. These dyed fabrics also demonstrated that they had decent deodorization and antibacterial activity [220].

Cotton was treated by padding with persimmon juice and its dyeability and functionality was assessed, an assessment that included deodorization ratio and antibacterial activity. The advantages of padding-based dyeing are many, but of note is the easier reproduction of color over traditional hand dyeing where it is possible to find that various colors and color fastness to light and laundering are not easy to obtain. By padding in larger numbers, dyed fabrics displayed red-yellow colors that were deeper, with low brightness and chromatic colors that were high. These cotton fabrics that had been dyed had a 4~5 perspiration fastness rating, a 3~4 rubbing fastness rating, and a washing fastness with a rating of 4. As padding numbers and sunlight exposure time or UV light increases, colors gradually became considerably deeper, with the color development completed around 70 h of UV exposure. Among the sources of light, the UV light exposed dyed fabrics demonstrated a deeper yellow-red color than those that were exposed to sunlight. Treating cotton fabrics with premordants such as Sn, Cr, Cu, and Al demonstrated strong yellow colors, particularly those fabrics that were treated with the Fe mordant, which demonstrated greenish red-yellow colors. As persimmon juice padding times of dyeing increased, properties such as stiffness and water repellency were enhanced correspondingly in the warp and weft directions. The fabrics dyed in this process also demonstrated as having good antibacterial activity and deodorization [221].

In this context, the environmental protection becomes clear, given the reduction in chemicals used to dye fabrics. Moreover, the water-repellent property enhancement with this natural treatment makes the use of plastics to protect from water less necessary. If we are to think in terms of clothes and other applications, this functionality could be important. Even more so, antibacterial activity and deodorization are especially important in the developed world given the health and social impact that these features among societies are sought after in various personal care and hygiene products. A lot of dermo cosmetics trades seek these two properties for their products. However, even more, increasingly societies tend to look for these characteristics in products made from natural compounds, and as such, these works demonstrate the added value of using persimmon juice in the fabric industry.

#### 1.3.4. Plant Growth Regulation

Persimmon fruit powder [0.5%, 1.0%,2.5% and 5.0% (*w/v*)] and Indolbi, a synthetic plant growth regulator, as a candidate to boost nutritional value and the yield of soybean sprouts, were evaluated because of potential health hazards, given that synthetic chemical use like Indolbi is less preferred by consumers when the alternative can be found in natural products. The increment in the yield was nearly magnified in 2.5 and 5.0% compared to the Indolbi treated sprouts based on the control. Total phenolic contents, isoflavones, and vitamin C as well as antioxidant potentials were also significantly higher at 2.5% compared to the Indolbi treatment and the control group. Nonetheless, total free amino acid and magnesium contents of Indolbi treated sprouts was greater than those in the fruit powder treatments. In general, results of this research demonstrated that persimmon fruit powder is a viable green option for enhancing the nutritional value and yield of soybean sprouts [222]. More and different concentrations need to be tested as well as other cultures that could benefit from this treatment as was demonstrated by soybean sprouts, so it is important to expand this study.

#### 1.3.5. Biofuel Production

The particularities of Rojo Brillante persimmon, for instance, makes it necessary to find solutions for the valorization of this agro-food waste. Industrial persimmon residue may be a source of antioxidants and, especially, carotenoids. β-carotene and lycopene quantities in persimmon waste have been found to be higher than in the whole fruit, which is a remarkable result since this fruit is a good source of carotenoids. In contrast to extraction, which might constitute an expensive option, the use of a crude flour or powder from persimmon residue could represent a less expensive alternative and provide applications such as functional ingredients, natural preservative, flavoring, or coloring agents. On the other hand, according to the increasing concern in the reduction in indirect land use because of biofuel production and the need for the development of processes for the use of waste materials as a source for second generation bioethanol, persimmon residue has been assayed as a substrate for bioethanol production. Among the different processes assayed, the simultaneous saccharification and fermentation process resulted in higher ethanol yields and higher ethanol production based on the higher solid load, which implied more sugars available for fermentation, but reduced substrate inhibition phenomena. To produce bioethanol, waste from persimmon could be used alone or mixed with the residual biomass of other food. The recent results presented by Conesa and colleagues could be valuable in order to develop integrated approaches for the valorization of persimmon residue [223] and expand the bioethanol production to other byproducts such as persimmon waste, helping in the transition to clean sources of energy production and accelerate environmental decarbonization.

Reintroducing value-added compounds or products obtained from food waste materials into the economic cycle is of capital importance for the present food industry, given not only the environmental cost and waste management expenses, but also the potential economic and social benefits of the valorization process [223].

#### 1.3.6. Dermocosmetic Applications

Tannins in persimmon extract could be used as a promising whitening agent, which could improve the pigmentation and uneven distribution caused by ultraviolet radiation with the help of energy-based devices, which can lead to skin conditions such as freckles and melasma. Arbutin was a known drug for the treatment of skin pigmentation, but in a study performed by Sun and colleagues, 20% of persimmon tannin extract was better than arbutin in inhibiting pigmentation. The work suggests that persimmon tannins could be added to cosmetics as a functional ingredient. This work in the area of dermatology provides a research basis for the medical application and dermatological treatment with products based on persimmon tannins. This recent study added theoretical guidance for improving the economic added value of persimmons, expanding its industrial development, and developing high-processed persimmon products with high added value [120]. Given the evidence for *Diospyros kaki*’s phytotherapeutic effects and given the very narrow product scale on the market, this is all very encouraging for the development of future health care products. Even if toxicity assessment studies on herbal products and natural plants are few and far between [224], more studies and scale-up are required.

#### 1.3.7. Nanotechnology

Bioavailability primarily depends upon the degree of polymerization and the low bioavailability of proanthocyanidins, which significantly limit the correlated health effects [149].

Due to high molecular weight and a great capacity of reaction with other components of the digestive tract, proanthocyanidins have low accessibility and bioavailability [225]. To improve the bioavailability of proanthocyanidins, one apoferritin cage could approximately encapsulate 25.6 molecules of the proanthocyanidin dimer. The research demonstrated that the encapsulation of the proanthocyanidin dimers safeguarded it from temperature and oxidant damage, higher efficiency in transport was achieved, and these nanoparticles had superior cellular antioxidant activity. The novel strategy provided by Zhang and colleagues’ study indicated that protein cage structures like ferritin show promise in their application to the field of food nutrition [226]. Encapsulated proanthocyanidins based on casein-maltodextrin Maillard conjugates enhanced the bioaccessibility of proanthocyanidins, prolong lifespan of *Caenorhabditis elegans* by upregulating SOD and CAT activities, and antioxidant activity of proanthocyanidins was protected by encapsulation during storage and heat [227].

**Figure 2:**
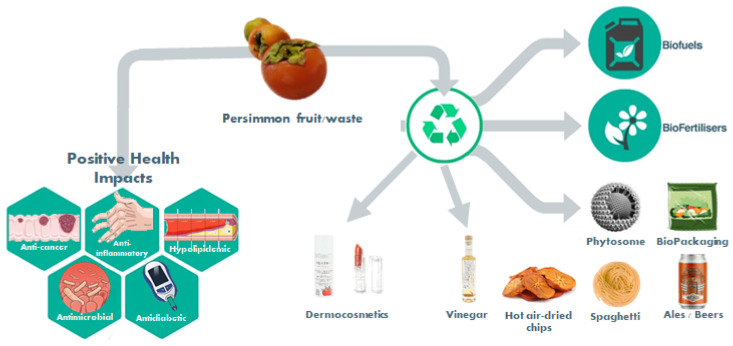
Solutions from the reinvention of old products with persimmon fruit and the addition of byproducts.

However, no works of nanotechnology encapsulation of these molecules have used proanthocyanidins from *Diospyros kaki*, even if this kind of phenolic compound, which are so abundant in the fruit or even in the peel, is often considered as waste (Figure 2).

Only one green nanotechnology using a rich phenolic extract from this fruit has been applied to load nanocarriers: phytosomes. These delivery systems are promising contenders for the future delivery of the bioactive agents of persimmon fruit extracts given that the extract-loaded phytosomes under accelerated storage conditions over six months had greater antioxidant activity, not to mention that these phytosomes were also capable of encapsulating 97.4% of total phenolic compounds, protecting them from the harsh environment such ass heat and humidity and with the preliminary in vivo results for the demonstration of tolerability, it could be an interesting dosage form for nutraceutical purposes and food supplements. More research in this field is still required in order to increase diligence and strengthen the mechanisms of action as well as its chronic use [75].

Analyses of the global food system call for a rapid evolution toward human diets that are sustainable, but how this feat might be achieved within the current global food regime has unfortunately, to date, not been sufficiently explored. Moberg and colleagues examined the factors that have fostered major dietary shifts across eight countries in the past 70 years, and their analysis proposes that this much-anticipated sustainability transition will necessitate intervention from the public and private-sector as well as consumers who value and can afford more sustainable and healthier diets [228].

For their part, scientists are actively engaging in sustainability strategies in order to intensify production to tackle environmental crises and food security [229]. In the same way that the COVID-19 pandemic demands an integrated, cooperative, and global response, in which science plays an essential role, so does feeding the global population [230], embracing the preservation of the Earth and environmental protection.

## 2. Conclusions

Given that persimmon is a very perishable and seasonal fruit, to increase availability to consumers and increase persimmon’s (*Diospyros kaki* L.) nutritional and/or functional value, new products should be developed. The scientific studies about these kinds of applications suggest that the edible part of fruit, only the peel, or even the pedicel could offer new possibilities for powders, flours, or juice to be included in food formulations to improve the content of bioactive compounds and its bioaccessibility; produce semi-finished products such as juices, purees, and pulps prepared with better storage stability; hot-air-drying of persimmon slices for dried chip production; beer; vinegar; emulsifier; anti-*Helicobacter pylori* agents; agents to control the spread and infection of methicillin-resistant *Staphylococcus aureus* in foods; biodegradable and edible films used as a lid for minimally processed vegetables packaging; eco-friendly alternative reductant for dyes; skin whitening agent; green plant growth regulator; and bioethanol production are some of the already achieved pilots of persimmon fruit and persimmon waste applications. Reintroducing value-added compounds or products obtained from food waste materials into the economic cycle is adding value to the cultivar while at the same time preserving the environment. Diversity of options is the key; there is no single solution that will guarantee sustainable nutrition for everyone, neither environmental protection against food waste nor disequilibrated agricultural production for worldwide food production.

Further research is needed regarding all the potential new products presented in this review. Obviously, scaling-up is fundamental to pass the responsibility onto the people to work daily toward attaining a sustainable transition. In the cases discussed related to the health impact of persimmon extracts or compound administration, mechanistic studies and clinical trials are fundamental, and the application of in vitro data with the help of in silico, as demonstrated with the usage of molecular docking methodology, is promising for the solution of problems presented and discussed in this review, but the use of some knowledge presented here to apply to highlight other issues is also fundamental in the context of sustainable nutrition and environmental emergency.

Solutions come from the reinvention of old products with the addition of byproducts from persimmon or with new products based on persimmon fruit and its byproducts, or with new and better packaging for the preservation of food products with postharvest technologies to preserve and extend the shelf-life of food products such as persimmon fruit. Given the present context worldwide, reintroducing added-value compounds or products obtained from food waste materials into the economic cycle is of capital importance for the current food industry, given not only the environmental cost and waste management expenses, but also the potential economic and social benefits of this valorization process. Beyond the world food crisis and climate emergency, new and better day-to-day solutions are needed right now. It has become clear that the uses for persimmon go far beyond the kitchen table.

It is impossible to ignore that the desired transition to sustainability will demand active public policy leadership as well as private-sector technological innovation in concert with all those consumers who culturally value (and can pay for) healthy and sustainable diets. Considerably more work will be needed from all stakeholders to reach this goal.

## Figures and Tables

**Table 1:** Generic nutritional composition of persimmon [34,35].

Parameters	100 g of the Edible Part
Energy (Kcal)	71.50
Proteins (g)	0.64
Total lipids (g)	0.25
Carbohydrates (g)	17.30
Fibers (g)	2.60
Water (g)	80.86
Calcium (mg)	8.00
Iron (mg)	0.20
Magnesium (mg)	9.25
Zinc (mg)	0.11
Sodium (mg)	2.50
Potassium (mg)	230.00
Phosphorus (mg)	19.50
Selenium (µg)	0.60
Thiamine (mg)	0.03
Riboflavin (mg)	0.03
Niacin equivalents (mg)	0.20
Vitamin B6 (mg)	0.10
Vitamin C (mg)	11.75
Total Vitamin A (retinol equivalents) (µg)	177.00
Folate (µg)	7.00

**Table 2:** Main phenolic compounds from persimmon fruits quantified and identified.

Phenolic Compounds (PCs)	Quantification and Reference			
Gallic acid (mg/100 g FW)	0.953 ± 0.344 [20]	2.794 ± 0.263 [75]	2.789 ± 0.003 [76]	2.43 ± 0.215 [26]
Caffeic acid (mg/100 g FW)	0.078 ± 0.001 [76,77]	0.1 ± 0.001 [75,76]		
P-coumaric (mg/100 g FW)	0.048 ± 0.004 [76]	0.097 ± 0.004 [75]	0.088 ± 0.046; 0.113 ± 0.055 [20]	
Ferulic acid (mg/100 g FW)	0.1 ± 0.001 [75,76]	0.008 ± 0.003 [20]		
Chlorogenic acid (mg/100 g FW)	0.171 ± 0.016 [75]	0.274 ± 0.003 [76]		
Protocatechuic acid (mg/100 g FW)	0.013 ± 0.010; 0.004 ± 0.002 [20]	0.005 ± 0.000 [75]		
Ellagic acid (mg/100 g FW)	0.327 ± 0.173 [20]			
Quercetin (mg/100 g FW)	0.224 ± 0.002;0.812 ± 0.006 [76]			
Proanthocyanins (mg/100 g FW)	540.2 ± 0.000 [74]	744 ± 8.6 [75]		
**Identifications**				
(Epi)catechin and (epi)gallocatechin	[13,59,70,71,72,82]			
Quercetin 3--2′′-galloylglucoside), quercetin 3-*O*-glucoside and isomer and aglycone	[59]			
Kaempferol-3-*O*-glucoside, kaempferol 3-(2′′-galloylglucoside)	[59]			
2-Methoxy-1, 4-benzoquinone	[59]			

**Table 3:** Some scientific works in vitro, in vivo, and in humans demonstrating biological effects related to *Diospyros kaki* L and *D. Lotus* L.

Target	Biological Effect (s)	References
Atherosclerosis	Male Wistar rats and male mice (C57BL/6.Cr) submitted to a high-cholesterol diet showed that fruit administration made it difficult to increase lipid levels in serum and made it difficult to decrease antioxidant activity in plasma. Rat diets enriched with either 7% of phenol-free dry persimmon or 7% whole dry persimmon enhanced lipid levels. This was considerable when entire dry persimmon was included. Persimmon’s antioxidant effect was primarily linked with its phenols and was attained through the addition of whole dry fruit to basal diet. The capacity of dried young persimmon fruit to bind bile acid adds to its hypolipidemic effect in mice, and tannins are one of the functional constituents in young persimmon fruit.	[73,91,111]
Lipidic metabolism	With male C57BL/6 mice, bile acid-binding ability of kaki-tannin was analyzed versus cholic acid, deoxycholic acid, glycocholic acid, and taurocholic acid in vitro. The impact on fecal bile acid excretion in mice was also analyzed. Kaki-tannin’s bile acid-binding ability was feebler than that of cholestyramine, all the bile acids analyzed were adsorbed by kaki-tannin and considerably fostered fecal bile acid excretion in mice when given at 1% (*w/w*) in the diet. Kaki-tannin was able to bind bile acids, within the range of concentrations of bile acids found in the human intestine. The cultivars of young persimmon (Fuyu and Hachiya) administered to rats induced a significant reduction in the levels of total cholesterol, low-density lipoprotein (LDL), and triglycerides in relation to the control and the group of the mature persimmon (also of the Fuyu and Hachiya type). It prevented high-fat diet induced liver steatosis. Expression of liver cholesterol 7 alpha-hydroxylase gene (CYP7A1) was increased by approximately three times. Highly polymerized tannins were the functional constituents related to the hypolipidemic effect demonstrated. Hypolipidemic impacts of young persimmon fruit on apolipoprotein E-deficient C57BL/6.KOR-ApoEshl mice fed a diet supplemented with dry young persimmon fruit. This treatment considerably decreased plasma chylomicron, very low-density lipoprotein (VLDL) and low-density lipoprotein (LDL) cholesterols, and triglyceride, and this reaction was complemented by an increase in the excretion of fecal bile acid. Within the liver, sterol regulatory element binding protein-2 gene expression was considerably greater in mice fed young persimmon fruit, while the mRNA and protein levels of the LDL receptor were unaltered. These findings suggest that increase in the speed of fecal bile acid excretion is a key mechanism of the hypolipidemic impact induced by young persimmon fruit in C57BL/6.KOR-ApoEshl mice. Fermented persimmon extract (FPE) is completely unknown. The impacts of FPE on mice metabolic parameters fed with a high-fat diet (HFD) was analyzed. Results showed that supplementation with FPE led to an approximate 15% body weight decrease, abdominal and liver fat decrease, and lowered serum levels of total cholesterol, triglycerides, and glucose. FPE was also found to hinder the differentiation of murine 3T3-L1 pre-adipocyte cells into mature adipocytes. It is suggested that gallic acid is a key bioactive element of FPE, and that AMP-activated protein kinase facilitates the positive impacts of FPE and gallic acid. Kaki-tannin adsorbed all the bile acids analyzed and meaningfully encouraged the excretion of fecal bile acid within the range of concentrations of bile acids found in the human intestine in male mice C57BL/6J when supplied at 1% (*w/w*) in the diet, proving useful in the prevention and amelioration of metabolic syndrome.	[18,93,112,113]
	Male Sprague-Dawley rats were fed a 2% high-cholesterol diet and given distinct doses of high molecular weight persimmon tannin (HMWPT) or without HMWPT for 9 weeks. A treatment of 100 mg high molecular weight tannins/kg of body weight per day could significantly increase the activity of serum lecithin cholesterol acyl transferase (LCAT) and fecal excretion of bile acids. The deposition of hepatic lipid droplets and hepatic steatosis, prompted by the high cholesterol diet, were clearly hampered by high molecular weight tannins. HMWPT was accountable for the hypocholesterolemic impact of persimmon and it may well exert a hypolipidemic impact by stimulating serum LCAT activity, boosting fecal bile acid excretion and enhancing antioxidant profile.	[114]
Glucose Metabolism/Type 2 Diabetes (T2D)	Expression of genes associated with fatty acid synthesis and glycolysis was increased, while gene expression associated with β-oxidation and gluconeogenesis was decreased in the liver of Goto-Kakizaki rats fed a diet supplemented with the extract of persimmon peel. Apoptosis-related gene expression decreased, while ribosome-related gene expression was increased in the group of rats that ingested persimmon peel extract. Results showed that the insulin signaling pathway was triggered in the persimmon peel group, with a subsequent increase in insulin sensitivity. Ingestion of this extract helped to maintain euglycemia and lipid homeostasis. Fat-soluble extract from persimmon peel and fed T2D Goto-Kakizaki (GK) rats an AIN-93G rodent diet enhanced with persimmon peel extract (PP diet) for 12 weeks. In contrast with the control AIN-93G diet, the PP diet considerably lowered the activity of plasma glutamic-pyruvatetransaminase, with buildup of β-cryptoxanthin in the liver. DNA microarray assessments showed that the persimmon peel diet modified hepatic gene expression profiles. Expression of insulin signaling pathway-related genes was considerably enhanced in differentially expressed gene sets. Western blotting analysis also demonstrated a rise in insulin receptor beta tyrosine phosphorylation in PP diet fed rats. Persimmon peel extract impacts gene expression related to the insulin signaling pathway, the PP diet increases insulin resistance in GK rats. Downregulation of *Ptpσ* through administration of persimmon peel extract promotes tyrosine phosphorylation of IRβ, leads to activation of the insulin signaling pathway, and upregulates genes related to both glucose homeostasis and lipid homeostasis. Results indicate that dietary intake of persimmon peel extract can assist in maintaining euglycemia.	[1]
Chronic inflammation of collagen-induced arthritis (CIA)	Persimmon extract’s anti-inflammatory activity in rats with collagen-induced arthritis (CIA) was demonstrated by the considerable decrease in both the edema volume and radiological variations credited to bone CIA. Administering persimmon extract (15 mg/kg p.o. per day) diminishes the extent of chronic inflammation and tissue damage typical of CIA in rats, effect related most likely to the potent antioxidant qualities of the extract. Taking into consideration the repressive effect of the fresh persimmon fruit extract (1.1–17.5 μg/mL) on human neutrophil oxidative burst, an IC_50_ of 7.5 ± 1.0 μg/mL was established. In both of these cases, the extract showed the ability to act as an intracellular antioxidant as well as the capability to interfere with the neutrophil function, hindering the release of deleterious reactive oxygen species that would magnify the inflammatory signals already activated. The valuable properties of persimmon extract in this model might be associated with a reduction in neutrophil activation and subsequent decrease in the release of proinflammatory neutrophil-derived products, which is also in harmony with the positive results seen in the paw edema models (induced by CIA and carrageenan), both of which represent inflammation models characteristically with high correlation to neutrophil activation. The extract proved capable of acting as an intracellular antioxidant as well as inhibiting the neutrophils’ function, constraining the release of deleterious ROS that amplified inflammatory signals already triggered.	[54]
Allergic inflammation	Mast cell-mediated allergic inflammation in vivo, systemic anaphylaxis model was induced in mice that were given an intraperitoneal injection [8 mg/kg of body weight (BW)] of the mast cell degranulator, compound 48/80 and passive cutaneous anaphylaxis (PCA) model in mice were induced and in vitro studies of histamine and β-hexosaminidase levels, cAMP, and intracellular calcium levels, real-time polymerase chain reaction (RT-PCR) to analyze the mRNA expression of TNF-α, IL-1β, and β-actin, nuclear and cytosolic p65 NF-κB, and IκBα were assayed using anti-NF-κB (p65) and anti-IκBα antibody. The persimmon extract repressed the release of histamine and β-hexosaminidase from mast cells by modulating intracellular calcium levels; diminished gene expression and the secretion of the pro-inflammatory cytokines, tumor necrosis factor (TNF)-α, and interleukin (IL)-1β was obtained by inhibiting nuclear factor-kB, effects comparable to those observed with disodium cromoglicate, suggesting the potential therapeutic use of a *Diospyros kaki* aqueous extract in allergic inflammatory disorders. AEDK constrains systemic allergic reaction and the delivery of histamine in serum, which is an index of mast cell degranulation. Additionally, administering AEDK to mice kept them from IgE-mediated PCA, one of the most significant in vivo models of acute local anaphylaxis. In systemic and local anaphylaxis, the repressive effects of AEDK were similar to those of DSCG, a clinically used medication for treating asthma and allergies. AEDK restrains the delivery of calcium from intracellular calcium stores, suggesting the regulatory role of cAMP in histamine release. Due to the structural similarity of EGCG and catechin, it was theorized that catechin may be one of the compounds accountable for the anti-allergic and anti-inflammatory effects of AEDK.	[31]
Sarcopenia (age-related syndrome characterized by progressive loss of mass and strength of skeletal muscles)	An extract of *Diospyros kaki* was tested using a Caco-2 cell coculture system. An in vitro model for studying the toxicity and metabolism of drugs including bioactive compounds of plants. Caco-2 cells subjected to 0.5 mg/ml *D. Kaki* extract diminished the oxidative stress-induced decline of mouse myoblast cell C2C12 viability, which indicates that the extract may be able to promote intestinal epithelial cells to produce secretions that decrease oxidative stress in myoblasts in vitro. This feature is linked to the ability of persimmon extracts to stimulate epithelial cells to produce secretions that possess the ability to inhibit intracellular ROS production. The concept of functional substances is highlighted here. The effect of the Japanese persimmon fractions tested on C2C12 growth activity was found to be different in a single culture and coculture system. This result showed that the metabolic product from Caco-2 cell culture treated with this fraction had a considerable impact on C2C12 cell viability. The effects of phytochemical compounds and cellular communications that may be involved in the metabolism of the test compounds is critical in assessing the bioavailability of tested compounds. DPPH method, ORAC assay, total phenolic content (TPC), and trans-endothelial electrical resistance (TEER) were measured for measuring electrical resistance of a cell, and ROS intracellular production quantification by the oxidation-sensitive fluorescent probe 2′,7′-dichlorodihydrofluorescein diacetate (H2DCFDA) was performed.	[115]
Cognition deficits and oxidative damage in senescent mice	High molecular weight persimmon condensed tannin (HMWPT) meaningfully augmented the decreased activities of superoxide dismutase, catalase, raised the lowered total anti-oxidation capability, glutathione (GSH), and hydroxyproline contents, and reduced the raised monoamine oxidase, total cholinesterase activities, and malondialdehyde level in serum, liver, or brain of senescent mice, an aging mice model induced by D-galactose in a dose-dependent fashion. Additionally, HMWPT substantially diminished the D-galactose induced number decline, neuronal degeneration, and karyopycnosis in cells in the hippocampus and reduction in thickness of skin epidermis and dermis. This accounted for the amelioration of the spontaneous behavior and cognitive performance and skin aging inhibition. HMWPT may well decrease memory impairment and enhance behavior performance in the D-gal induced aging mice.	[116]
*In vitro* cerebral ischemia	Persimmon extract protected PC12 cells from oxidative stress generated by the deprivation of glucose, oxygen, and serum (Glucose-Oxygen-Serum Deprivation (GOSD)-Induced PC12 Cells Injury) via an antioxidant mechanism, with reduced intracellular ROS production. The intracellular ROS levels was accomplished with a fluorescent probe, H2DCF-DA, and the effects of PeHE and PuHE on ROS production following GOSD insult in PC12 cells revealed that pretreatment (2 h) with peel and pulp fruit extracts of *D. kaki* was able to encourage cell survival and reduce ROS growth upon GOSD stress in PC12 cells. A potential system behind the mitigation of ROS production following ischemic insult is due to antioxidant and free radical scavenger characteristics of persimmon.	[117]
Cancer	Nine human cancer cell lines (A375, A549, ACHN, C32, caco-2, COR-L23, Huh-12, LNCaP, and MCF-7) were tested with *D. Lotus* extract as well as eight compounds (quercetin, kaempferol, methylgallate, ellagic acid, gallic acid, myricetin, myricetin 3-*O*-α-ramnoside, and myricetin 3-*O*-β-glucuronide) isolated from the *D. Lotus* fruit. *D. lotus* extract tested in different in vitro systems (ABTS, DPPH, FRAP, and Fe2+ chelating activity assay) demonstrated considerable antioxidant activity. *D. lotus* extract exhibited high antioxidant activity and chelating properties, and these activities are related to the phenolic content. The extract exerted the greatest antiproliferative activity of the cells of the tumor line COR-L23, among the hydrolyzed tannins identified, ellagic acid showed strong antiproliferative activity against C32 and A375 cells. Gallic acid showed the highest cytotoxic activity against Caco-2 cells.	[118]
Melanoma	The prepared extract of the skin of the Japanese persimmon (*Diospyros kaki Fuyu*) inhibited the melanin biosynthesis in the B16 rat melanoma cells. From this extract, two active compounds were isolated, which were identified as flavonoid glycosides, isoquercitrin (quercetin-3-*O*-glucoside) and hyperine (quercetin-3-*O*-galactoside). These two glycosylated flavonoids showed a strong inhibitory effect on melanin production, this inhibitory effect was due to the suppression of tyrosinase protein expression and not on tyrosinase activity.	[119]
Inhibition of melanogenesis (skin whitening effects)	The guinea pig pigmentation model was established by ultraviolet B (UVB) irradiation (with a height of 10 cm from the skin and ultraviolet intensity of 1395 UW/cm^2^, each for 24 min per day for a total of 5 days). Half male and half female white guinea pigs were used. Masson–Fontana silver staining was employed to examine the impacts of persimmon tannin extract on melanin distribution in guinea pigs’ skin tissue and arbutin as a positive control. Tyrosinase activity was also assessed, and an enzyme-linked immunosorbent assay was employed to examine the contents of antioxidant enzymes, inflammatory factors, and signaling pathway inhibitors in the guinea pigs’ skin tissue. The persimmon (*Diospyros Kaki* Thunb.) tannin extract (the medium-dose group—20% persimmon tannin extract) could significantly reduce melanin density in white guinea pigs. The variations in experimental results were statistically significant (*p* < 0.01). The medium-dose group was more effective in inhibiting tyrosinase activity than arbutin. IL-6 and IL-8 expression were decreased by around 22.2% and 54%. Inhibition of inflammatory mediators can reduce melanogenesis in melanocytes. In contrast with the model group, catalase, glutathione peroxidase, superoxide dismutase, persimmon tannin extract (PTE) could significantly increase the content of CAT, GPX, and SOD in skin tissues after UVB irradiation. This reduces the level of active oxygen in melanocytes and prevents the activation of melanin synthesis. The Wnt/β-catenin signaling pathway can promote melanogenesis, while DKK1 might inhibit the binding of Wnt protein to its receptor. The effect of PTE in the medium-dose group was better than that in the arbutin group, and the DKK1 content was 8% higher than that in the arbutin group. Overexpression of DKK1 could significantly inhibit cell survival and melanogenesis. In conclusion, PTE could inhibit melanocyte growth and strongly inhibit melanogenesis, which could inhibit skin pigmentation caused by UVB irradiation. The optimal content of persimmon tannin for inhibiting pigmentation was 20%. The inhibitory tyrosinase activity was raised by 24.3%, 33.3%, 59.3%, 36.81%, and 17.16%, respectively. By now, the health-promoting potential of the tannins is widely recognized; however, the application of persimmon tannin in whitening and inhibiting pigmentation has rarely been examined and reported.	[120]
Human lymphoid leukemia Molt 4B cells	A persimmon extract and a few of its individual phenolic compounds (epicatechin gallate, epigallocatechin, epicatechin, and catechin) were studied on the growth of human lymphoid leukemia Molt 4B cells, and it was observed that the extract as well as epigallocatechin and epicatechin gallate reduced the growth of these cells in a dose dependent manner. After treating for 3 days, severe cell damage was observed such as DNA fragmentation. Apoptosis of these cells were induced by tested phenolic compounds.	[81]
Thyroid cancer	A case-controlled analysis of thyroid cancer in Korean women, emphasized an inverse correlation observed between eating persimmons and benign and malignant thyroid cancer risk. Elevated consumption of raw vegetables, tangerines, and persimmons might reduce the risk of developing thyroid cancer and assist in the prevention of early-stage thyroid cancer.	[121]
Colitis and colon cancer cell	A phenolic-rich extract was tested using an in vivo model (male CD-1 mice) of experimental colitis (TNBS-induced colitis) and an in vitro model of colon adenocarcinoma cells (HT-29). Results demonstrated beneficial effects of a phenolic extract of persimmon in the reduction in experimental colitis severity, reduction of diarrhea severity, hemorrhagic injury, the reduction of the mortality rate, and the successful impairment of cell proliferation and invasion in HT-29 cell model. A reduced expression of iNOS and COX-2 in the colonic tissue of colitis mice contributes to the impairment of the inflammatory process in the colon. There was no inhibition of the gelatinase MMP-9 and MMP-2 activities, something which could partly result from the colitis animal model, where the phenolic concentrations utilized were far more diminished than those necessary to reduce MMP-9. The NF-κB pathway is a potential aim for the beneficial effects of the persimmon phenolic extract, as evidenced in these experiments. Considering the part that inflammatory processes play in the progression of CRC and the significant link between inflammation and cancer, the results highlight the potential of persimmon phenolic compounds as a pharmacological tool in the treatment of IBD patients. Persimmon phenolic extract may well be acting pleiotropically in several mechanisms of action and/or acting on an upstream mediator of inflammatory processes that reduce the expression of COX-2 and iNOS.	[17]

## Data Availability

Not Applicable.
